# IL-27 receptor-regulated stress myelopoiesis drives abdominal aortic aneurysm development

**DOI:** 10.1038/s41467-019-13017-4

**Published:** 2019-11-06

**Authors:** Iuliia O. Peshkova, Turan Aghayev, Aliia R. Fatkhullina, Petr Makhov, Elizaveta K. Titerina, Satoru Eguchi, Yin Fei Tan, Andrew V. Kossenkov, Marina V. Khoreva, Lyudmila V. Gankovskaya, Stephen M. Sykes, Ekaterina K. Koltsova

**Affiliations:** 1 Blood Cell Development and Function Program, Fox Chase Cancer Center, Philadelphia, Pennsylvania 19111 USA; 2 Pirogov Russian National Research Medical University, Moscow, 117997 Russia; 3 Cancer Biology Program, Fox Chase Cancer Center, Philadelphia, Pennsylvania 19111 USA; 4 Lewis Katz School of Medicine, Temple University Cardiovascular Research Center, Philadelphia, Pennsylvania 19140 USA; 5 Genomics Facility, Fox Chase Cancer Center, Philadelphia, Pennsylvania 19111 USA; 6 Bioinformatics Facility, The Wistar Institute, Philadelphia, Pennsylvania 19104 USA

**Keywords:** Interleukins, Haematopoietic stem cells, Aneurysm

## Abstract

Abdominal aortic aneurysm (AAA) is a prevalent life-threatening disease, where aortic wall degradation is mediated by accumulated immune cells. Although cytokines regulate inflammation within the aorta, their contribution to AAA via distant alterations, particularly in the control of hematopoietic stem cell (HSC) differentiation, remains poorly defined. Here we report a pathogenic role for the interleukin-27 receptor (IL-27R) in AAA, as genetic ablation of IL-27R protects mice from the disease development. Mitigation of AAA is associated with a blunted accumulation of myeloid cells in the aorta due to the attenuation of Angiotensin II (Ang II)-induced HSC expansion. IL-27R signaling is required to induce transcriptional programming to overcome HSC quiescence and increase differentiation and output of mature myeloid cells in response to stress stimuli to promote their accumulation in the diseased aorta. Overall, our studies illuminate how a prominent vascular disease can be distantly driven by a cytokine-dependent regulation of bone marrow precursors.

## Abstract (web summary)

Immune cells contribute to the aortic wall destruction during abdominal aortic aneurysm (AAA) development. Here, Peshkova et al. show that cytokine signaling through interleukin-27 receptor is required for Angiotensin II-induced myelopoiesis and mature myeloid cells production, thus contributing to their aortic accumulation and aneurysm progression

## Introduction

Abdominal aortic aneurysm (AAA) is a cardiovascular disease (CVD) characterized by abdominal aorta dilatation caused by accumulated immune cells, inflammation, and degradation of the medial layer followed by aortic rupture and bleeding, which is often fatal^1–4^. The current standard of care for AAA is limited to surgery at the later stages of disease progression. Thus, a better understanding of its mechanisms, particularly the inflammatory nature of AAA, is urgently needed. Various risk factors are associated with AAA pathogenesis^5^, including elevated blood pressure that is mediated by activation of the renin–angiotensin system (RAS) and upregulation of Angiotensin II (Ang II). Accordingly, a model of AAA that employs long-term infusion of Ang II in atherosclerosis-prone mice recapitulates many aspects of human AAA, including elevated blood pressure, dependence on higher Ang II levels, and gradual recruitment of immune cells^6,7^. Moreover, the disease in this model is exacerbated by hypercholesterolemia, one of the additional risk factors for AAA^8^. The importance of RAS/Ang II in connection between blood pressure and AAA in humans is supported by clinical trials data where blood pressure-reducing medications targeting RAS/Ang II circuit significantly decreases the incidence of aortic dissection^9^. However, Ang II acts via AT1a receptors (AT1aRs), which are broadly expressed on different cell types in the aortic wall, and therefore “hypertension-independent” functions of Ang II in AAA have also been widely observed^10–14^.

Chronic inflammation presented as infiltration of various immune cells is a key feature and driver of AAA^2,4,15,16^. Neutrophils, monocytes, and macrophages are bone marrow (BM)-derived myeloid cells that play important roles in immunity and tissue repair^17^. These cells also contribute to the aortic inflammation and vessel destruction associated with AAA^4,18–20^. Their output from hematopoietic stem and progenitor cells (HSPCs) in the BM can be influenced by environmental stimuli and stresses. During infection or inflammatory insult, a variety of cytokines and danger-associated molecular patterns stimulate HSPCs in BM to rapidly increase the production and systemic release of innate immune cells^21–23^. Interestingly, HSPCs are also responsive to Ang II^24^, which drives their expansion in mice^25^. Although alterations in myelopoiesis have been reported to play a role in AAA progression^4,18^, the key cytokine signaling factors that control HSPC fate and hematopoiesis in AAA have yet to be determined. Particularly, the contribution of Ang II and cytokine signaling in the regulation of HSPC proliferation and myeloid cell differentiation to AAA pathogenesis has not been examined.

Interleukin (IL)-27 is a member of IL-6/IL-12 cytokine superfamily that regulates various hematopoietic and non-hematopoietic cells in infectious diseases and autoimmunity^26–31^. The role of IL-27 signaling in AAA and its contribution to BM cell production in response to chronic stress stimuli has not been investigated.

Here we demonstrate that genetic inactivation of IL-27 receptor (IL-27R) protects mice from AAA. Mechanistically, we show that IL-27R signaling is essential to drive HSPC proliferation, differentiation, and BM output. These findings illustrate how IL-27R signaling acts distantly to control AAA development by cooperating with stress-induced factors in the BM to accelerate stress hematopoiesis and promote the production of myeloid cells that are subsequently recruited to the aortic wall mediating its destruction.

## Results

### IL-27R signaling promotes Ang II-induced AAA development

Inactivation of IL-27R exacerbates atherosclerosis^29–31^ and leads to the development of certain abdominal aortic lesions, which typically are rare in atherosclerotic mice. As atherosclerosis and AAA share some common underlying chronic inflammatory mechanisms^5^, we initially investigated whether the lesions in IL-27R-deficient mice may be an incipient source of AAA formation, and that IL-27R deficiency would increase inflammation and promote AAA.

**Fig. 1:**
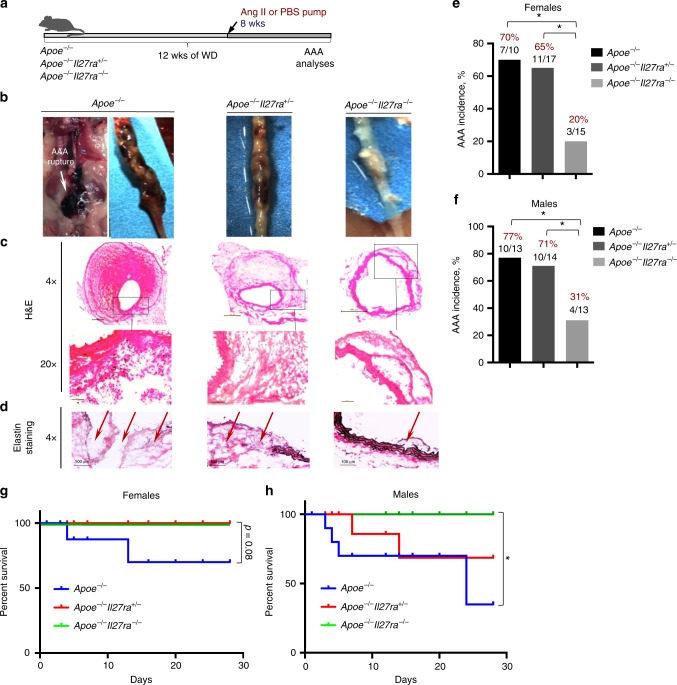
IL-27R deficiency protects from Ang II-induced AAA development. **a** Scheme of the experiment. *Apoe*^*−/−*^, *Apoe*^*−/*^^*−*^*Il27ra*^*+/*−^ and *Apoe*^*−/−*^*Il27ra*^*−/−*^ female and male mice were fed the WD for overall period of 12 weeks, and during last 4 weeks of feeding they were implanted with osmotic pumps containing Ang II or PBS. **b** Representative images of suprarenal aortas with developed AAA. **c** Hematoxylin and eosin (H&E) staining, **d** Verhoeff-Van Gieson staining of AAA frozen sections from *Apoe*^*−/−*^, *Apoe*^*−/−*^*Il27ra*^*+/*−^, or *Apoe*^*−/−*^*Il27ra*^*−/−*^ mice after Ang II infusion. Scale bars, 100 μm. Black-elastin, red-collagen, blue-nuclei. Arrows indicate ruptured elastic lamina. **b**–**d** Representative images from male mice. **e**, **f** AAA incidence among *Apoe*^*−/−*^ (*n* = 10), *Apoe*^*−/−*^*Il27ra*^*+/−*^ (*n* = 17), and *Apoe*^*−/−*^*Il27ra*^−*/−*^ (*n* = 15) female (**e**) and *Apoe*^*−/−*^ (*n* = 13), *Apoe*^*−/−*^*Il27ra*^*+/*−^ (*n* = 14), and *Apoe*^*−/−*^*Il27ra*^*−/−*^ (*n* = 13) male mice (**f**). *p < 0.05, Fisher’s exact test (two-sided). **g**, **h** Survival curves for *Apoe*^*−/−*^ (2**/**10 died*)*, *Apoe*^*−/−*^*Il27ra*^*+/−*^ (0/17 died), and *Apoe*^*−/−*^*Il27ra*^*−/−*^ (0/15 died*)* female (**g**) and *Apoe*^***−/−***^ (4/13 died), *Apoe*^*−/−*^*Il27ra*^*+/−*^ (2/14 died), and *Apoe*^*−/−*^*Il27ra*^*−/−*^ (0/13 died*)* male mice (**h**) during 28 days of Ang II infusion. *p < 0.05, long-rank test. Data are mean ± SEM from at least three independent experiments

To evaluate the potential role of IL-27R signaling in AAA, we first employed a well-characterized mouse model of AAA driven by chronic Ang II infusion (800 ng kg^−1^ min^−1^) using a surgically implanted osmotic mini-pump into mice on *Apoe*^*−/−*^ background^6,32^. To exclude any differences in genetics or microbiota, we used cage-mate and littermate controls. As hypercholesterolemia promotes AAA development^8,33^, male and female *Apoe*^*−/−*^, *Apoe*^*−/−*^*Il27ra*^*+/−*^ or *Apoe*^*−/−*^*Il27ra*^*−/−*^ mice were fed a Western diet (WD) for 8 weeks followed by Ang II pump implantation. Four weeks later, mice were assessed for abdominal aorta bulging and AAA development (Fig. 1a). Ang II infusion induced AAA formation in IL-27R-proficient *Apoe*^*−/−*^ and *Apoe*^*−/−*^*Il27ra*^*+/−*^ mice, whereas unexpectedly the incidence of AAA was markedly reduced in IL-27R-deficient *Apoe*^*−/−*^ mice (Fig. 1b-f). Blood pressure was elevated in response to Ang II infusion, but IL-27R regulated AAA independent of effects on blood pressure. Body weight also remained unchanged by IL-27R deficiency (Supplementary Fig. 1a, b). Both male and female *Apoe*^*−/−*^ and *Apoe*^*−/−*^*Il27ra*^*+/−*^ mice developed larger AAAs with visual hemorrhages in the artery wall compared with their *Apoe*^*−/−*^*Il27ra*^*−/−*^ counterparts (Fig. 1b, c and Supplementary Fig. 1c). Verhoeff-Van Gieson staining showed extensive disruption and degradation of elastic lamina in the aortas of both *Apoe*^*−/−*^ and *Apoe*^*−/−*^*Il27ra*^*+/−*^ mice, but not in *Apoe*^*−/−*^*Il27ra*^*−/−*^ mice (Fig. 1d). Female *Apoe*^*−/−*^ and *Apoe*^*−/*−^*Il27ra*^*+/−*^ mice (Fig. 1e) developed slightly lower rates of AAA than did their male counterparts (Fig. 1f); however, the incidence of AAA was reduced by IL-27R deficiency in both genders (Fig. 1e, f). Although both *Apoe*^*−/−*^ and *Apoe*^*−/−*^*Il27ra*^*+/−*^ control mice experienced significant sudden AAA-related mortality in the Ang II model, 100% of *Apoe*^*−/−*^*Il27ra*^*−/−*^ mice remained alive throughout the experiment (Fig. 1g, h). Pathological severity index, which was calculated based on the level of aortic wall degradation and immune infiltrate^34^, showed that both female and male *Apoe*^*−/−*^ and *Apoe*^*−/−*^*Il27ra*^*+/−*^ mice displayed more advanced stages of AAA (IV stage) compared with IL-27R-deficient *Apoe*^*−/−*^ mice, where AAA progression, if any, was restricted to the early stages (I–II) (Supplementary Fig. 1d). The effect of IL-27R deficiency on AAA development was also confirmed in another AAA model^35^ induced by topical application of elastase combined with administration of 0.2% β-aminopropionitrile (BAPN) in drinking water (Supplementary Fig. 2).

To ensure that our observations were not restricted to the dietary factors introduced by the WD, we also performed experiments on a cohort of mice fed a regular chow diet. As expected, incidence of AAA was significantly lower in *Apoe*^*−/−*^ and *Apoe*^*−/−*^*Il27ra*^*+/−*^ male mice fed the chow diet compared with WD-fed groups and AAA formation was not detected at all in females fed with chow diet. However, IL-27R deficiency still rendered male mice to be less susceptible to AAA induction (Supplementary Fig. 3). Collectively, our data demonstrate that IL-27R signaling promotes AAA in two distinct in vivo models of AAA.

### IL-27R signaling controls myeloid cells accumulation in AAA

**Fig. 2:**
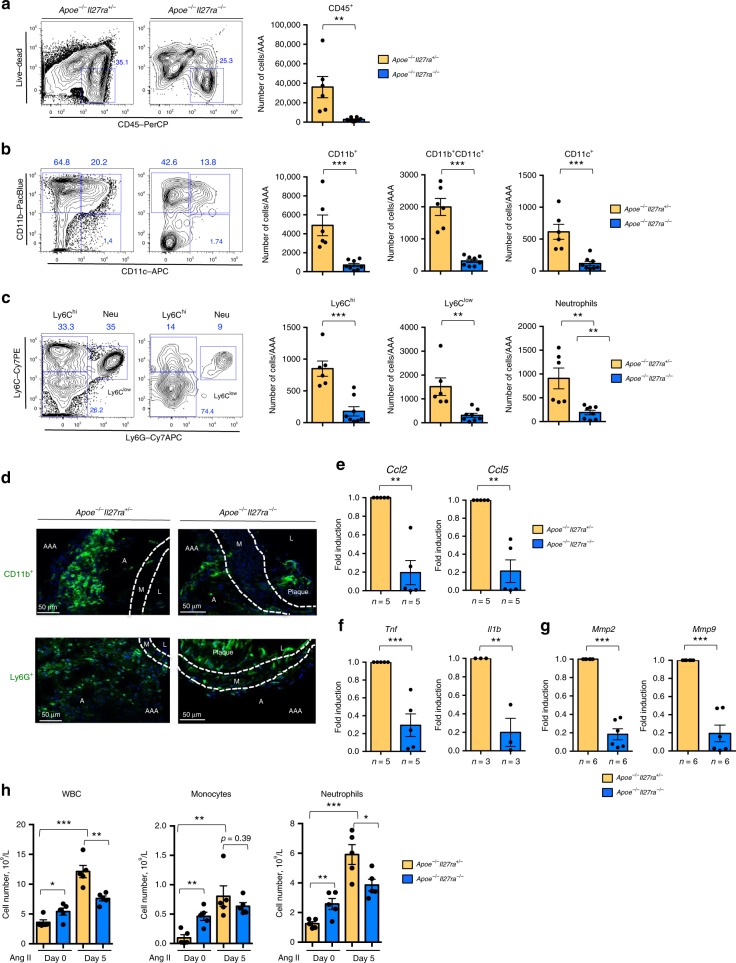
Limited accumulation of myeloid cells in AAA of IL-27R-deficient mice. Single-cell suspensions of suprarenal aortas/AAA of *Apoe*^*−/−*^*Il27ra*^*+/−*^ (*n* = 6) or *Apoe*^*−/−*^*Il27ra*^*−/−*^ (*n* = 8) mice fed with WD and infused with Ang II were stained for Live/Dead, CD45, CD11b, CD11c, Ly6G, Ly6C, and analyzed by FACS. **a** Left: representative FACS plots of CD45^+^ cells. Numbers indicate percentage of cells in each gate. **a** Right**:** absolute number of live CD45^+^ cells. **b** Representative FACS plots and absolute number of CD45^+^ live CD11b^+^, CD11b^+^CD11c^+^, and CD11c^+^ cells. **c** Representative FACS plots and absolute number of live CD11b^+^Ly6C^hi^, CD11b^+^Ly6G^+^ (Neutrophils), and CD11b^+^Ly6C^low^ cells. **p < 0.01, ***p < 0.005, unpaired Student’s *t*-test (two-tailed). **d** Immunofluorescent confocal analysis of localization and abundance of CD11b^+^ and Ly6G^+^ (Neutrophils) cells in suprarenal aortas/AAA of *Apoe*^*−/−*^*Il27ra*^*+/−*^ and *Apoe*^*−/−*^*Il27ra*^*−/−*^ mice infused with Ang II. L-lumen, M-media, A-adventitia, AAA-abdominal aortic aneurysm. Representative images from three independent experiments. Relative gene expression of chemokines (**e**), cytokines (**f**), and MMPs (**g**) in suprarenal aortas/AAA of *Apoe*^−*/−*^*Il27ra*^*−/−*^ mice were normalized to *Rpl32* expression and then normalized to gene expression in control *Apoe*^*−/−*^*Il27ra*^*+/−*^ mice. **p < 0.01, ***p *<* 0.005, Wilcoxon’s signed-rank test. **h** Leukocytes, monocytes, and neutrophils counts were evaluated by VetScan HM5 in the blood of *Apoe*^*−/−*^*Il27ra*^*+/−*^ (*n* = 5) or *Apoe*^*−/−*^*Il27ra*^*−/−*^ (*n* = 5) mice prior to and on day 5 after pump implantation. Data are mean ± SEM from at least three independent experiments. *p < 0.05. **p < 0.01, ***p < 0.005, unpaired Student’s *t*-test (two-tailed)

AAA progression is associated with increased accumulation of various immune cells at the site of vessel injury^2,4^. Flow cytometry analysis of isolated and digested suprarenal aortas revealed a significant reduction in the percentage and number of hematopoietic CD45^+^ cells in *Apoe*^*−/−*^*Il27ra*^*−/−*^ mice compared with *Apoe*^*−/−*^*Il27ra*^*+/−*^ controls (Fig. 2a and Supplementary Fig. 4). Among CD45^+^ cells, the number of CD11b^+^, CD11b^+^CD11c^+^, and CD11c^+^ myeloid cell subsets were also significantly diminished in aortas of *Apoe*^*−/−*^*Il27ra*^*−/−*^ mice (Fig. 2b). We observed a striking reduction in monocyte subsets (Ly6C^hi^ and Ly6C^low^) as well as neutrophils (Ly6G^+^) in AAA lesions of *Apoe*^*−/−*^*Il27ra*^*−/−*^ mice compared with IL-27R-sufficient controls (Fig. 2c). Immunofluorescence staining of isolated AAAs confirmed limited adventitial accumulation of CD11b^+^ myeloid cells, particularly Ly6G^+^ neutrophils in AAA lesions of *Apoe*^*−/−*^*ll27ra*^*−/−*^ mice (Fig. 2d). Quantitative reverse-transcriptase PCR (Q-RT-PCR) analysis revealed the reduction of *Ccl2* and *Ccl5* chemokines involved in attraction and tissue trafficking of myeloid cells, as well as *Tnf*, *Il1b*, and matrix metalloproteinases (MMPs) involved in inflammation (Fig. 2e–g). The analysis of circulating immune cells showed that Ang II infusion heightened the number of circulating leukocytes, especially neutrophils in IL-27R-sufficient but not IL-27R-deficient mice (Fig. 2h).

Thus, Ang II infusion promoted the expansion and accumulation of IL-27R-sufficient myeloid cells in suprarenal aorta. In contrast, IL-27R deficiency limited the expansion of circulating cells and reduced the accumulation of myeloid cells, particularly monocytes and neutrophils in AAA, affecting the expression of myeloid-derived chemokines, cytokines, and key enzymes in the area of AAA formation.

### IL-27R signaling regulates Ang II-induced HSPC expansion

**Fig. 3:**
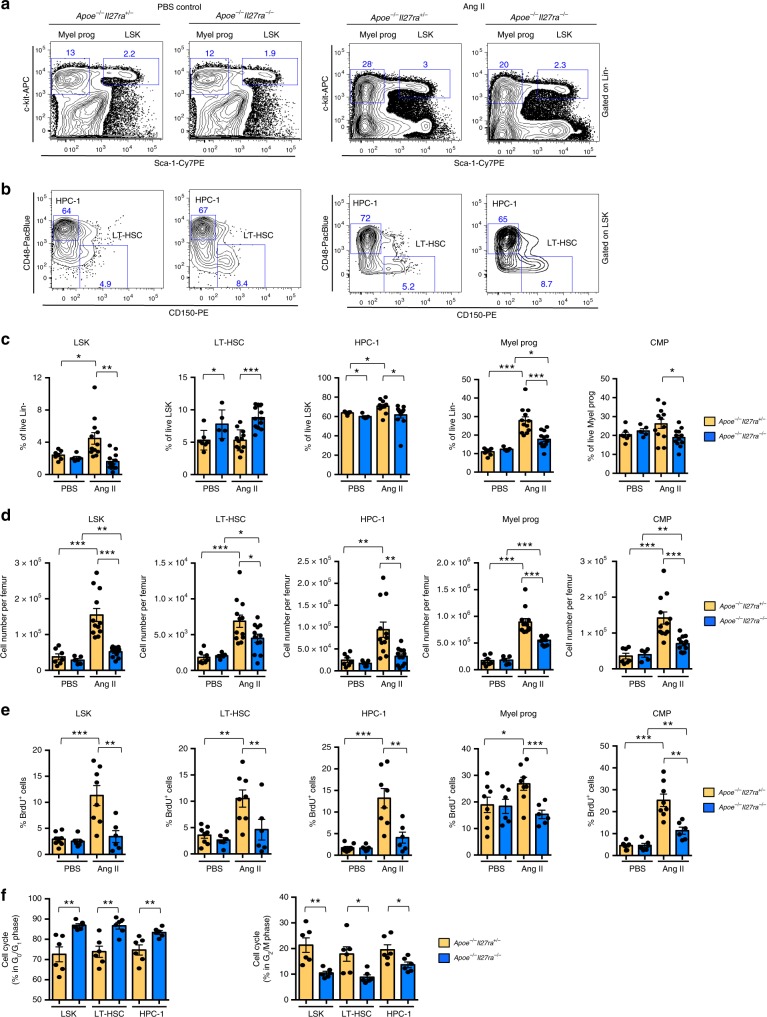
IL-27R deficiency leads to limited expansion of HSCs in response to Ang II. BM was obtained from *Apoe*^*−/−*^*Il27ra*^*+/−*^ (*n* = 7) and *Apoe*^*−/−*^*Il27ra*^*−/−*^ (*n* = 5), or *Apoe*^*−/−*^*Il27ra*^*+/−*^ (*n* = 12) and *Apoe*^*−/−*^*Il27ra*^*−/−*^ (*n* = 12) mice fed with WD for overall period of 12 weeks and infused with PBS or Ang II for last 4 weeks of feeding, respectively. **a** Representative dot plots of live LSK (Lin^−^Sca-1^+^c-kit^+^) and myeloid progenitors (Lin^−^Sca-1^−^c-kit^+^). **b** Representative dot plots of live LT-HSC (Lin^−^Sca-1^+^c-kit^+^CD150^+^CD48^−^) and HPC-1 (Lin^−^Sca-1^+^c-kit^+^CD48^+^CD150^−^) cells. **c** Average percentage of live LSK, LT-HSC, HPC-1, myeloid progenitors, and CMP (Lin^−^Sca-1^−^c-kit^+^CD16/CD32^−^CD34^+^). **d** Absolute number of live LSK, LT-HSC, HPC-1, myeloid progenitors, and CMP. **e** Proliferation of HSPCs from *Apoe*^*−/−*^*Il27ra*^*+/−*^ (*n* = 8) or *Apoe*^*−/−*^*Il27ra*^*−/−*^ (*n* = 6) mice fed with WD and infused with Ang II or PBS as determined by BrdU incorporation. Percentage of live BrdU-positive LSK and myeloid progenitors, including LT-HSC, HPC-1, and CMP. Data are mean ± SEM from two independent experiments. **f** Percentage of progenitor cells from *Apoe*^*−/−*^*Il27ra*^*+/−*^ (*n* = 6) or *Apoe*^*−/−*^*Il27ra*^*−/−*^ (*n* = 6) mice infused with Ang II in G_0_/G_1_ and G_2_/M phase of cell cycle, analyzed for BrdU inclusion, and DAPI staining. Data are mean ± SEM from three independent experiments. *p < 0.05, **p < 0.01, ***p < 0.005, unpaired Student’s *t*-test (two-tailed)

Inflammation associated with infection and injury is known to modulate mobilization and output of hematopoietic cells from the BM^23,36^. Next, we sought to determine whether the reduced myeloid cell accumulation in AAA sites of suprarenal aortas and limited number of circulating cells mediated in IL-27R-deficient mice is due to alterations in hematopoiesis. We analyzed the cellular composition of BM isolated from *Apoe*^*−/−*^*Il27ra*^*−/−*^ and control *Apoe*^*−/−*^*Il27ra*^*+/−*^ mice. In steady state (mice infused with phosphate-buffered saline (PBS)), IL-27R deficiency did not cause dramatic changes in LSK population (Lin^−^Sca-1^+^c-kit^+^) or myeloid progenitors (Lin^−^Sca-1^−^c-kit^+^) including common myeloid progenitors (CMP: Lin^−^Sca-1^−^c-kit^+^CD16/CD32^−^CD34^+^) (Fig. 3a, c, d and Supplementary Fig. 5a). The percentage of long-term progenitors (LT-HSC: Lin^−^Sca-1^+^c-kit^+^CD150^+^CD48^−^) was slightly elevated, whereas the percentage of short-term progenitors (HPC-1^37^, also referred as MPP3/4:^38^ Lin^−^Sca-1^+^c-kit^+^CD150^−^CD48^+^) population was slightly reduced in IL-27R-deficient mice (Fig. 3b–d), a phenomenon associated with increased HSC quiescence^39,40^. In agreement with previous observations^25^, Ang II infusion caused the expansion of multiple LSK populations, including LT-HSC, HPC-1, and total myeloid progenitors, including CMP in IL-27R-sufficient mice compared with PBS-treated non-stressed controls. Strikingly, IL-27R deficiency blunted the Ang II-driven expansion of LSK, LT-HSC, HPC-1, myeloid precursors, and CMP (Fig. 3a–d).

In line with previous observations that Ang II receptor (AT1aR) is expressed on HSPCs^24^ and Ang II infusion promotes both proliferation and “myeloid-biased” differentiation of HSCs^25^, we found that Ang II infusion strongly increased the proportion of proliferating cells, marked in vivo by bromodeoxyuridine (BrdU) incorporation, in IL-27R-sufficient but not -deficient animals, although PBS-infused mice did not display any significant differences (Fig. 3e and Supplementary Fig. 5b). Cell cycle analysis showed higher proportion of IL-27R-deficient cells in G_0_/G_1_ and less in G_2_/M phase of cell cycle compared with IL-27R-sufficient cells (Fig. 3f). These data suggest that IL-27R signaling is required to potentiate the proliferative effect of Ang II on HSPCs and is involved into the regulation of HSPC cell cycle.

**Fig. 4:**
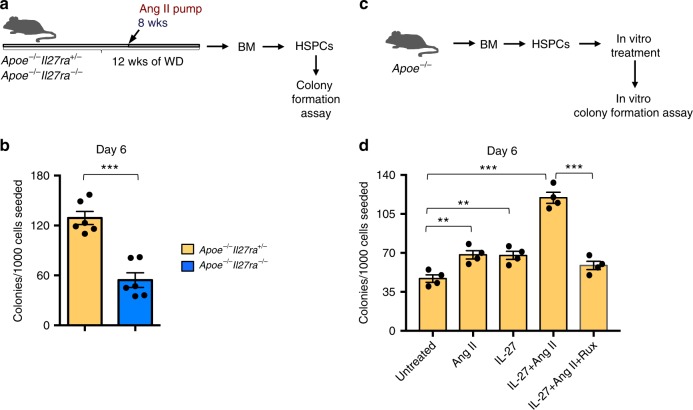
IL-27R signaling is needed for HSPC differentiation. **a** Scheme of ex vivo colony-formation experiment. **b** Lin^−^ HSPCs were isolated from BM of *Apoe*^*−/−*^*Il27ra*^*+/−*^ (*n* = 6) or *Apoe*^*−/−*^*Il27ra*^*−/−*^ (*n* = 6) mice infused with Ang II and plated in M3434 media under myeloid conditions. Granulocyte–monocyte (GM) colony formation was accessed on day 6. **c** Scheme of in vitro colony-formation experiment. **d** Lin^−^ HSPCs were isolated from BM of *Apoe*^*−/−*^ (*n* = 4) mice and plated in M3434 media under myeloid conditions in the presence of Ang II, recIL-27, Ruxolitinib, or their combination. GM colony formation was accessed on day 6. Data are mean ± SEM from three independent experiments. **p < 0.01, ***p < 0.005, unpaired Student’s *t*-test (two-tailed)

To evaluate the effect of IL-27R signaling on the clonogenic and differentiation capacities of HSPCs, we performed ex vivo colony-formation assays. Lineage-depleted (Lin^−^) BM HSPCs (Supplementary Fig. 5c) isolated from *Apoe*^*−/−*^*Il27ra*^*−/−*^ and *Apoe*^*−/−*^*Il27ra*^*+/−*^ mice that had been infused with Ang II for 4 weeks were cultured in M3434 media under myeloid differentiation conditions. We found that IL-27R-deficient HSPCs showed a marked decrease in total numbers of colonies formed compared with IL-27R-sufficient cells (Fig. 4a, b). Conversely, in vitro recombinant (rec) IL-27 stimulation promoted myeloid colony formation, especially when combined with Ang II (Fig. 4c, d). Importantly, pharmacological inhibition of JAK1/2 kinases, which are immediately downstream of IL-27R, with ruxolitinib ablated this stimulating effect (Fig. 4d).

Overall, these data suggest that IL-27R signaling potentiates the Ang II-induced HSPC proliferation and differentiation, and “stress” myelopoiesis during AAA development.

### IL-27R-deficient HSCs fail to induce AAA

**Fig. 5:**
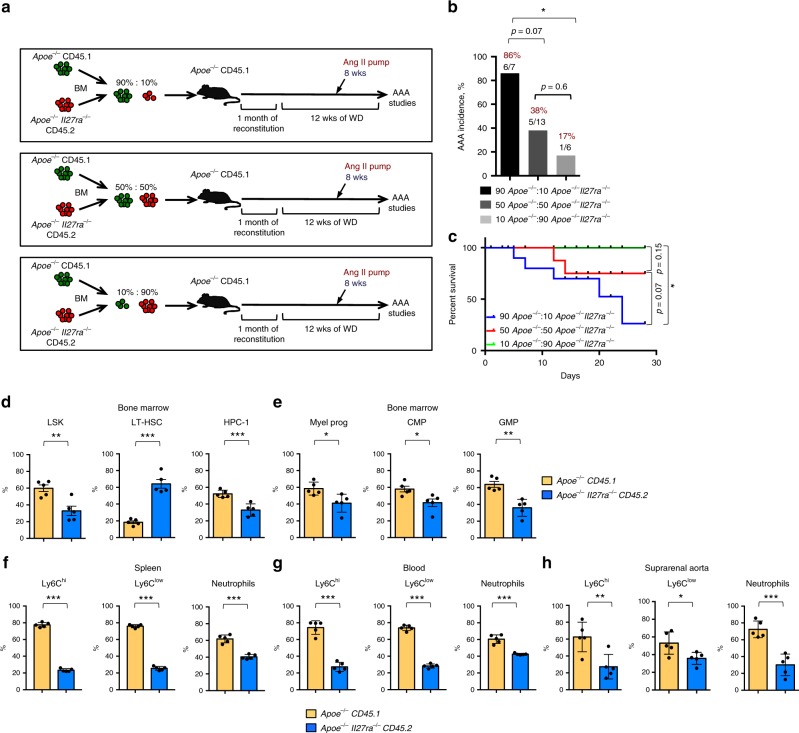
IL-27R deficient HSCs fail to induce AAA. **a** Scheme of experiment. *Apoe*^*−/−*^ CD45.1-recipient mice were lethally irradiated and reconstituted with donor mixes of *Apoe*^*−/−*^ CD45.1 and *Apoe*^*−/−*^*Il27ra*^*−/−*^ CD45.2 total BM cells in a 90%:10%, 50%:50%, and 10%:90% ratio, respectively. Four weeks after reconstitution mice were placed on WD for 12 weeks and infused with Ang II for last 4 weeks of feeding. **b** Percentage of AAA incidence among *Apoe*^*−/−*^ CD45.1-recipient mice receiving BM mixture in a ratio: 90%*Apoe*^*−/−*^*:*10%*Apoe*^*−/−*^*Il27ra*^*−/−*^ (*n* = 7), 50%*Apoe*^*−/−*^*:*50%*Apoe*^*−/−*^*Il27ra*^*−/−*^ (*n* = 13), or 10%*Apoe*^*−/−*^*:*90%*Apoe*^*−/−*^*Il27ra*^*−/−*^ (*n* = 6). *p < 0.05, Fisher’s exact test (two-sided). **c** Survival curves for *Apoe*^*−/−*^ CD45.1-recipient mice receiving BM mixture in a ratio: 90%*Apoe*^*−/−*^*:*10%*Apoe*^*−/−*^*Il27ra*^*−/−*^ (5/7 died), 50%*Apoe*^*−/−*^*:*50%*Apoe*^*−/−*^*Il27ra*^*−/−*^ (2/13 died) or 10%*Apoe*^*−/−*^*:*90%*Apoe*^*−/−*^*Il27ra*^*−/−*^ (0/6 died) during 28 days of Ang II infusion. *p < 0.05, long-rank test. Proportion of live donor-specific cells in *Apoe*^*−/−*^ CD45.1-recipient mice (*n* = 5) receiving BM mix in 50%*Apoe*^*−/−*^*:*50%*Apoe*^*−/−*^*Il27ra*^*−/−*^ ratio. **d** LSK cells, including LT-HSC and HPC-1; **e** myeloid progenitors, including CMP and GMP (Lin^−^Sca-1^−^c-kit^+^CD16/CD32^+^CD34^+)^ in the BM. Proportion of donor-specific mature Ly6C^hi^, Ly6C^low^-monocytes, and Ly6G^+^ (neutrophils) in the spleen (**f**), blood (**g**), and suprarenal aorta (**h**). Data are mean ± SEM from three independent experiments. *p < 0.05, **p < 0.01, ***p < 0.005, unpaired Student’s *t*-test (two-tailed)

Due to the inherent variability of AAA model, the effect on BM hematopoiesis may be mediated by direct role of IL-27R signaling or influenced by the size and incidence of AAA lesion development, thereby warranting studies where IL-27R-proficient and -deficient cells co-exist within the same animal. To evaluate whether a cell-intrinsic lack of IL-27R signals render HSPCs unable to expand and contribute to AAA in an overall IL-27R-sufficient environment, we conducted competitive BM transplantation (BMT) analyses. BM cells isolated from congenically marked *Apoe*^*−/−*^ CD45.1^+^ (further referred as wild type (WT)) or *Apoe*^*−/−*^*Il27ra*^*−/−*^ CD45.2^+^ (further referred as *Il27ra*^*−/−*^) mice were mixed in different ratios including 90%:10%, 50%:50%, and 10%:90%, respectively, and transplanted into lethally irradiated *Apoe*^*−/−*^ CD45.1^+^ IL-27R-sufficient recipients. Mice were placed on WD for 4 weeks after BM transfer and maintained on WD for another 8 weeks, allowing for a total of 12 weeks of BM reconstitution. The efficiency of introduction of correct BM mixes was confirmed by fluorescence-activated cell sorting (FACS) for CD45.1/CD45.2 ratio in the blood of naive unchallenged mice 4 weeks after BMT (Supplementary Fig. 6a). After 8 weeks of WD feeding, mice were implanted with Ang II pump to induce AAA and assess the effect of Ang II on the BM cells of different IL-27R status and their role in AAA (Fig. 5a). Interestingly, 86% of mice transplanted with a 90%wt:10%*Il27ra*^*−/−*^ mix of BM developed late stages of AAA and some died due to AAA rupture, whereas only 17% of mice transplanted with a 10%wt:90%*Il27ra*^*−/−*^ mix of BM developed AAA, arguing for cell autonomous pathogenic role of IL-27R. The incidence of AAA in mice transplanted with mix of 50%wt:50%*Il27ra*^*−/−*^ BM was 38% (Fig. 5b, c). Chimeric recipient mice, which received 50%wt:50%*Il27ra*^*−/−*^ BM mix and infused with Ang II, were used to compare side by side the development and accumulation of IL-27R-sufficient and IL-27R-deficient myeloid cells in IL-27R-sufficient environment. Both WT and *Il27ra*^*−/−*^ donors cells exhibited ~50% cell ratio in the peripheral blood 4 weeks after transplantation and before the Ang II infusion and AAA induction (Supplementary Fig. 6a). However, the analysis of HSPCs compartment in the BM of chimeric mice infused with Ang II revealed the reduction of *Il27ra*^*−/−*^ LSK, HPC-1, and more mature myeloid progenitors compared with WT-derived cells within the same mouse (Fig. 5d, e). Conversely, the percentage of LT-HSC derived from *Il27ra*^*−/−*^ BM was elevated (Fig. 5d), consistent with more quiescent state of IL-27R-deficient cells. In line with limited competitive expansion of IL-27R-deficient precursors, the percentage of mature peripheral *Apoe*^*−/−*^*Il27ra*^*−/−*^ (CD45.2)-derived monocytes and neutrophils were also diminished in the spleen and blood in comparison with IL-27R-sufficient *Apoe*^*−/−*^ (CD45.1) cells from the same recipient mice (Fig. 5f, g). Of note, the number of circulating myeloid cells of IL-27R-sufficient origin was elevated in response to Ang II infusion compared with PBS control, whereas significantly less prominent effect was observed in IL-27R-deficient cells (Supplementary Fig. 6b). The analysis of suprarenal aorta/AAA revealed preferential accumulation of monocytes and neutrophils derived from WT (IL-27R-sufficient) progenitors, whereas the percentage of accumulated cells derived from *Il27ra*^*−/−*^ BM was diminished (Fig. 5h and Supplementary Fig. 6c). This indicates that when WT and IL-27R-deficient BM progenitors compete in the same animals, WT myeloid cells are preferentially produced and therefore are overrepresented in AAA lesions. Importantly, no difference in cell trafficking/recruitment was observed between the genotypes once mature IL-27R-sufficient and IL-27R-deficient myeloid cells were administered intravenously (i.v.), thereby bypassing the stage of myeloid cell expansion in the BM (Supplementary Fig. 7). This indicates that primary defect of IL-27R deficiency is in their inability to properly expand in the BM and not in the recruitment of mature cells into the AAA site.

IL-27R deficiency was previously reported to promote atherosclerosis in *Apoe*^*−/−*^ mice^31^. Here we found that Ang II infusion accelerated the disease in IL-27R sufficient, but did not further increase atherosclerosis development in IL-27R-deficient mice (Supplementary Fig. 8a-c), eliminating the phenotypic difference between genotypes in Ang II-treated conditions. The analysis of myeloid cells accumulation in 50%wt:50%*Il27ra*^*−/−*^ BM chimeric mice revealed that myeloid cells in the aortic arch were mostly cells of WT *Apoe*^*−/−*^ (CD45.1) origin, whereas *Apoe*^*−/−*^*Il27ra*^*−/−*^ (CD45.2) cells were significantly underrepresented (Supplementary Figs. 6c and 8d). Thus, enhanced BM output of IL-27R-sufficient cells upon Ang II treatment drives augmented atherosclerosis, whereas Ang II is unable to cause the same enhancement of atherosclerosis in IL-27R-deficient hosts (Supplementary Fig. 8d). As a result, in Ang II-untreated conditions, *Apoe*^*−/−*^*Il27ra*^*−/−*^ mice develop more atherosclerosis in comparison with *Apoe*^*−/−*^*Il27ra*^*+/−*^ controls^31^. However, because of Ang II-driven increase in atherosclerosis in *Apoe*^*−/−*^*Il27ra*^*+/−*^ but not in *Apoe*^*−/−*^*Il27ra*^*−/−*^ mice, *Apoe*^*−/−*^*Il27ra*^*+/−*^, and *Apoe*^*−/−*^*Il27ra*^*−/−*^ Ang II-infused mice develop equally high levels of atherosclerosis.

Overall, our data suggest that Ang II-driven expansion of HSCs and myelopoiesis facilitates myeloid cell accumulation and AAA, and this process is dependent on IL-27R signaling.

### IL-27R regulates HSC quiescence genes in stress myelopoiesis

**Fig. 6:**
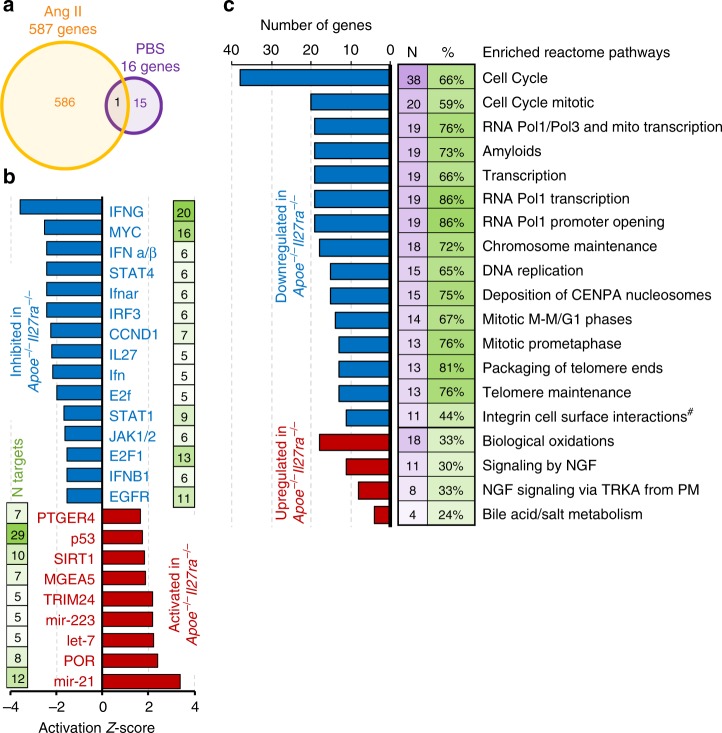
IL-27R regulates the quiescence of HSCs in Ang II-induced myelopoiesis. LT-HSCs were FACS-sorted from the BM of *Apoe*^*−/*−^(*n* = 2), *Apoe*^*−/−*^*Il27ra*^*+/−*^ (*n* = 4), or *Apoe*^*−/−*^*Il27ra*^*−/−*^ (*n* = 3) mice fed with WD for 12 weeks and infused with Ang II or PBS for last 2 weeks of feeding, and subjected to whole transcriptome analysis (RNA-seq). **a** Overall number of genes whose expression is changed between *Apoe*^*−/−*^ and *Apoe*^*−/−*^*Il27ra*^*−/−*^ mice either treated with Ang II (587 genes) or with PBS (control, 16 genes). **b** Deregulated upstream regulators whose targets were significantly overrepresented (*p* < 0.05, Fisher’s exact test) among genes uniquely affected by genetic IL-27R deficiency. **c** Various pathways significantly deregulated (*p* < 0.05, GSEA) by IL-27R deficiency, as determined by Reactome pathway enrichment analysis. Changed pathways include cell cycle, proliferation, and division in LT-HCS after Ang II infusion (^#^*p* = 0.056, GSEA)

To gain further insights into the mechanisms by which IL-27R signaling influences HSC function and “stress-induced” hematopoiesis in AAA, we performed whole transcriptome RNA-sequencing analysis of FACS-sorted LT-HSCs (Lin^−^Sca-1^+^c-kit^+^CD150^+^CD48^−^) and HPC-1 (Lin^−^Sca-1^+^c-kit^+^CD150^−^CD48^+^) isolated from the BM of *Apoe*^*−/−*^, *Apoe*^*−/−*^*Il27ra*^*+/−*^, or *Apoe*^*−/−*^*Il27ra*^*−/−*^ mice fed the WD and infused with PBS or Ang II for the last 2 weeks of the feeding. Consistent with our functional data in vivo and ex vivo, we found that the lack of IL-27R signaling does not significantly affect transcriptional profile of LT-HSCs in a “steady state” (PBS-infused mice), where only 16 genes were differentially expressed (false discovery rate (FDR) < 5%) between *Apoe*^*−/−*^*Il27ra*^*−/−*^ and *Apoe*^*−/−*^ LT-HSCs; however, Ang II infusion led to significant changes in gene expression, where 587 genes were differentially expressed between *Apoe*^*−/−*^*Il27ra*^*−/−*^ and *Apoe*^*−/−*^ LT-HSCs (FDR < 5%) (Fig. 6a). Interestingly, *Agr2*, an inhibitor of p53 pathway^41^, downregulated in *Apoe*^*−/−*^*Il27ra*^*−/−*^ LT-HSCs, was the only one gene that was differentially expressed in both PBS and Ang II-treated IL-27R-deficient HSCs (Fig. 6a). Only minor changes in transcriptional profile were found in HPC-1 subpopulation in response to Ang II infusion (Supplementary Fig. 9a, b). Using IPA Upstream Regulator Analysis for genes uniquely deregulated by Ang II in *Apoe*^*−/−*^*Il27ra*^*−/−*^ LT-HSCs as compared with *Apoe*^*−/−*^*Il27ra*^*+/−*^ LT-HSCs, we found 24 regulators with a significant number of their known targets (*p* < 0.05 by Fisher’s exact test, at least five significantly changed targets) overrepresented in the list of significantly affected by IL-27R deficiency genes whose combined behavior showed change in activation status of upstream regulator (Fig. 6b). The majority of the regulators that we found to be significantly activated or inhibited have been implicated into the control of HSC fate^40,42–50^. Two functional pathways in LT-HSCs were the most affected by IL-27R deficiency, namely (1) regulators of proliferation—Myc, CCND1, and E2F; and (2) pathways involved in IL-27R signaling and control HSC expansion—IFNα/β, IFNAR, STAT1, and IL-27 itself. Genes whose expression is controlled by aforementioned regulators in LT-HSCs are shown in a heatmap (Supplementary Fig. 9c). Notably, the p53 pathway had the most number of overrepresented targets in LT-HSCs of *Apoe*^*−/−*^*Il27ra*^*−/−*^ mice (29 targets, *p* = 2 × 10^−6^, Fisher’s exact test). Reactome pathway analysis by gene-set enrichment analysis (GSEA)^51^ identified multiple pathways including cell cycle and various pathways associated with cell division significantly downregulated in IL-27R-deficient LT-HSC (Fig. 6c).

These results suggest that distinct transcriptional profile regulated by IL-27R signaling is required to maintain the balance between quiescence, proliferation and differentiation in early hematopoietic precursors challenged with stress stimuli, as responsiveness of HSPCs to Ang II is blunted in the absence of IL-27R^39,40^. These data are consistent with our findings of reduced BrdU incorporation, cell accumulation in G_0_/G_1_ cell cycle phase, and limited BM output of IL-27R-deficient cells.

**Fig. 7:**
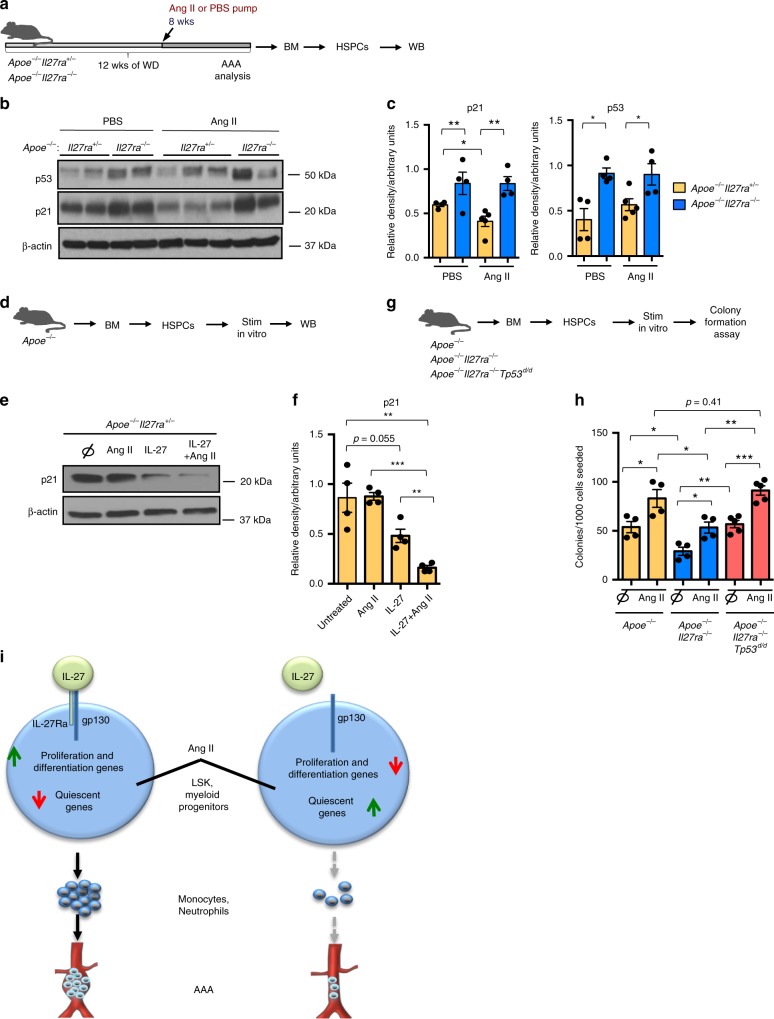
Elevated expression of p53 and p21 in IL-27R-deficient HSPCs. **a** Scheme of experiment. **b** Lin^−^ HSPCs isolated from WD-fed *Apoe*^*−/−*^*Il27ra*^*+/−*^ (*n* = 4) and *Apoe*^*−/−*^*Il27ra*^*−/−*^ (*n* = 4) mice infused with PBS or *Apoe*^*−/−*^*Il27ra*^*+/−*^ (*n* = 5) and *Apoe*^*−/−*^*Il27ra*^*−/−*^ (*n* = 4) mice infused with Ang II for 4 weeks were lysed and subjected to western blotting with p21, p53, and β-actin antibody. Each lane corresponds to individual mouse. **c** Quantification of WB analysis for p21 and p53, normalized to β-actin. **d** Scheme of experiment. **e** WB on Lin^−^ HSPCs from naive *Apoe*^*−/−*^*Il27ra*^*+/−*^ mice (*n* = 4) stimulated in vitro with Ang II, IL-27, or both for 24 h. **f** Quantification of WB analysis for p21. **g** Scheme of experiment. **h** Lin^−^ HSPCs from BM of *Apoe*^*−/−*^ (*n* = 4), *Apoe*^*−/−*^*Il27ra*^*−/−*^ (*n* = 4), or *Apoe*^*−/−*^*Il27ra*^*−/−*^*Trp53*^*d/d*^ (*n* = 5) mice were sorted and plated in M3434 media under myeloid conditions with or without Ang II. GM colony formation was accessed on day 6. Data are mean ± SEM from two independent experiments. *p < 0.05, **p < 0.01, ***p < 0.005, unpaired Student’s *t*-test (two-tailed). **i** Scheme describing the role of IL**-**27R signaling in regulation of Ang II-induced myelopoiesis and AAA. Elevation of Ang II in AAA provides a stimulus for the activation of “stress” myelopoiesis via regulation of gene expression controlling proliferation and differentiation in IL-27R-sufficient LT-HSCs. Lack of IL-27R signaling renders Ang II-primed cells unable to overcome quiescence state and significantly reduces proliferation of progenitors and myeloid cell bone marrow output, therefore reducing cell accumulation in AAA lesions and AAA progression

Along with the quiescence gene signature and upregulation of p53 gene targets uncovered by IPA Upstream Regulator Analysis, we found that IL-27R-deficient HSPCs sorted from Ang II-infused mice were characterized by elevated levels of p21/Waf1 protein, a prominent target of p53 pathway (Fig. 7a–c). To assess whether the observed effect on p21 expression was dependent upon Ang II and IL-27 action, we isolated HSPCs from *Apoe*^*−/−*^ mice and stimulated them in vitro with Ang II, recIL-27 or their combination (Fig. 7d). Indeed in vitro stimulation of HSPCs with Ang II and recIL-27 downregulated p21 protein levels (Fig. 7e, f). Interestingly, despite Ang II was required to stimulate HSPC proliferation, it was unable to induce downregulation of cell cycle inhibitors by itself and IL-27R signaling was essential to allow Ang II-stimulated progenitors to escape negative control of quiescence expression program and to start proliferation and differentiation.

Several signaling pathways could participate in the regulation of p21 expression, including interferon (IFN) and Myc pathways, both of which are enriched in Ang II-treated IL-27R-sufficient cells (Fig. 6b). In addition, p53 pathway, one of the key upstream bona fide regulators of p21 expression, was strongly affected by IL-27R deficiency (Fig. 6b). The analysis of protein expression revealed significantly higher p53 level in HSPCs from *Apoe*^*−/−*^*Il27ra*^*−/−*^ compared with *Apoe*^*−/−*^*Il27ra*^*+/−*^ mice infused with Ang II (Fig. 7a-c). To access whether cell proliferative effects of IL-27R signaling are mediated through the repression of p53-dependent gene transcription, we assayed colony formation of HSPCs with genetic ablation of *Trp53*. We found that genetic ablation of *Trp53* in IL-27R-deficient HSPCs restored their ability to form colonies similar to IL-27R-sufficient controls (Fig. 7g, h).

Finally, by Q-RT-PCR analysis we found that IL-27R-deficient HSPCs in vivo presented with the reduced expression of genes characteristic of myeloid lineages differentiation, expansion, and proliferation^40,42–45,52^ (Supplementary Fig. 10), demonstrating that not only cell cycle and proliferation of earlier progenitors is regulated by IL-27R signaling but also their ability to differentiate into myeloid lineages.

Collectively, these data suggest that HSPCs depends on IL-27R signaling to control the expression of various regulators of quiescence/proliferation balance, including p53-dependent regulators such as p21/Waf1. IL-27R signaling is required for cell cycle entry in response to Ang II-induced stimulation. This enhances myelopoiesis and BM myeloid cell output, overall contributing to AAA progression (Fig. 7i).

## Discussion

The function of IL-27R signaling was extensively investigated in various infectious models^27,28^ and an anti-inflammatory role IL-27R has been demonstrated in atherosclerosis^29–31^. Moreover, some of the IL-27R-deficient mice employed in the atherosclerosis studies developed small abdominal aorta lesions, grossly similar to potential incipient AAA sites, raising the possibility that IL-27R might also restrict spontaneous AAA development. The role of IL-27R in AAA, another vascular pathology with a clear role of yet unidentified inflammatory cytokine-driven mechanisms, however, has never been assessed. Here we made the unanticipated observation that mice with genetic ablation of IL-27R were largely protected from AAA. This correlated with diminished accumulation of monocytes and neutrophils in suprarenal aortas, which is a key early event in AAA^4,18–20^. The expression of myeloid cell-derived cytokines and chemokines was also decreased in the AAA lesions of *Apoe*^*−/−*^*Il27ra*^*−/−*^ mice infused with Ang II. Although homing mechanisms play an important role in the regulation of immune cell accumulation at the site of inflammation (AAA)^53^, the recruitment of a large amount of immune cells into rapidly developing AAA lesions may also require an increased BM output, known as “stress-induced” BM myelopoiesis. The unifying regulators required for the AAA development, vessel inflammation, and “stress myelopoiesis,” however, remained unknown. In earlier studies, Ang II was shown to induce the mobilization of splenic monocytes, whereas splenectomy and subsequent reduction of circulating monocytes suppresses AAA development^18^. Moreover, Ang II was shown to act directly on HSCs in BM and induce their self-renewal and rapid amplification of myeloid progenitors with subsequent production of mature myeloid cells^25^. In our model we found that Ang II causes the expansion of various HSCs and progenitor cells in the BM of IL-27R-sufficient (*Apoe*^*−/−*^ or *Apoe*^*−/−*^*Il27ra*^*+/−*^*)* mice, resulting in elevated numbers of circulating leukocytes. A surprising observation of our studies was, however, that inactivation of IL-27R signaling significantly blunted Ang II-driven expansion of HSCs and progenitor cells, and BM output, indicating a critical role of IL-27R signaling in potentiating Ang II-induced myelopoiesis, contributing to AAA development.

Several cytokines were shown to regulate the inflammatory milieu within the aortic wall in AAA^54–59^. However, the distant contribution of cytokines to AAA pathogenesis through systemic control of the immune response (e.g., through blood cell production due to hematopoietic stem cell (HSC) activation by stress factors (diet and high blood pressure tightly linked with elevated levels of Ang II) has not been previously explored. Here we establish IL-27R signaling as a critical regulator of Ang II-driven proliferation and differentiation of HSCs, essential for Ang II-induced stress myelopoiesis in a non-infectious, chronic vascular injury model. Given the role of myeloid cells in host defense and tissue repair^17^, this cytokine-driven induction of HSCs may be a common mechanism regulating the rapid need for increased BM output during various pathophysiological processes.

HSCs are maintained in a resting quiescent state in specialized hypoxic niches in the BM. In response to changes in microenvironment, they can exit this quiescent state and rapidly proliferate and differentiate into different blood cell types^23^. Maintenance of quiescence is crucial for preventing stem cell pool exhaustion, overproduction of mature cells, and the development of hematopoietic malignancies^60^. HSC quiescence is therefore tightly regulated by many factors including IFN cytokine signaling and miRNAs (miR-21, miR-223, and let-7)^44,61,62^. In chronic diseases, such as AAA, prolonged exposure to “stress” factors such as high-fat diet and Ang II affecting BM cells^25,63^ may provoke HSCs to exit quiescence and activate stress myelopoiesis, which serves as an additional pathogenic mechanism driving AAA. Here we establish IL-27R signaling as an in vivo regulator of HSC function in a non-infection model of vascular injury and AAA. This role of IL-27 is in line with the studies where IL-27 transgenic overexpression causes myeloproliferation in mice, and malaria infection drives the BM response and infection-induced myelopoiesis in a IFN/IL-27-dependent manner^64^.

By performing RNA-sequencing analysis of purified LT-HSCs we found that IL-27R-deficient LT-HSCs were characterized by a quiescence transcriptional profile, even following exposure to Ang II. Lack of IL-27R signaling rendered LT-HSCs unable to upregulate the expression of genes involved in cell division, proliferation, and differentiation normally induced by Ang II. Specifically, we found that the loss of IL-27R signaling in LT-HSCs most prominently inhibited pathways involved into the regulation of proliferation (Myc, E2F, and CCND1) and control of HSC expansion (IFN, STAT1). Conversely, inhibitors of proliferation were induced by IL-27R deficiency, particularly p53, miR-21, mir-223, and let-7^46–50^. Many of these regulators have also been previously implicated into the control HSCs fate. Indeed, miR-21 was shown to suppress hematopoiesis via activation of transforming growth factor-β (TGFβ) signaling^46^. MiR-223 has been also reported to attenuate hematopoietic cell proliferation^47^, whereas let-7 was shown to regulate HSC fate by controlling self-renewal, proliferation, quiescence, and differentiation via inhibition of TGFβ pathway^65,66^. Moreover, let-7 family members were shown to repress cell cycle regulators (Cyclin D1) and negatively regulate Myc expression^50,67^. These previous observations are in line with our transcriptomics results. TGFβ, p53, and IFN pathways may converge at the level of p21 expression, a key negative regulator of cell proliferation, whose levels in HSPCs are regulated by IL-27R signaling. In agreement with pronounced changes in transcriptional profile, *Apoe*^*−/−*^*Il27ra*^*−/−*^ HSCs demonstrated limited in vivo proliferation and cell cycle progression. Taken together, global gene expression profiling of LT-HSCs revealed that IL-27R signaling is required for HSC response to Ang II stimulation and potentiates stress-induced myelopoiesis. In the absence of this cytokine signaling, HSCs maintain quiescence even when AAA-inducing Ang II-driven stress is applied.

Collectively, our data establish an unexpected role of IL-27R signaling in AAA and suggest that Ang II, a stress factor that is constantly present in AAA, perhaps, in combination with other factors such as WD, promotes HSPCs proliferation and reveals a previously unexplored requirement for IL-27R signaling in Ang II-driven HSCs expansion and hematopoiesis during AAA development. Although our study is focused on the role of IL-27R signaling in hematopoietic compartment, IL-27R may also regulate AAA development via control of non-hematopoietic cells, representing an interesting subject for future work. Current work, however, strongly suggests that IL-27 can be a potential testable target for prevention and treatment of AAA and other forms of vascular injury, which require HSC BM mobilization for its full pathology. Therefore, overstimulation of this otherwise anti-inflammatory signaling pathway can actually be pathogenic in AAA due to its ability to modulate hematopoiesis.

## Methods

### Mice

I*l27ra*^*−/−*^ *(*JAX #018078*)* mice were crossed to *Apoe*^*−/−*^*(*JAX#002052*)* mice to obtain *Apoe*^*−/−*^, *Apoe*^*−/−*^*Il27ra*^*+/−*^, and *Apoe*^*−/−*^*Il27ra*^*−/−*^ mice. C57BL/6 CD45.1 (JAX#002014) mice were crossed to *Apoe*^*−/−*^ mice to obtain CD45.1 *Apoe*^*−/−*^ mice. All mice were on C57BL/6 background. Mice were bred and housed under specific pathogen-free conditions in an AAALAC-approved barrier facility at Fox Chase Cancer Center (FCCC). The genotyping was performed by standard PCR protocols. Animal numbers for each specific analysis are given in the figure legends. *Apoe*^*−/−*^, *Apoe*^*−/−*^*Il27ra*^*+/−*^, and *Apoe*^*−/−*^*Il27ra*^*−/−*^ mice were fed a WD (Teklad 88137) for 10 or 12 weeks beginning at 8 weeks after birth and subcutaneously implanted with Ang II containing pump for the last 2 or 4 weeks of feeding, after which mice were killed and AAA formation was analyzed. All animal experiments were approved by the Institutional Animal Care and Use Committee (IACUC) at FCCC and performed in compliance with all relevant ethical regulations for animal research.

### Models of AAA

*Ang II model*. Ang II containing osmotic mini-pumps were prepared and implanted as previously described^6^. Briefly, mice were anesthetized and osmotic mini-pumps (Alzet 2004, 10389–18) loaded with Ang II (800 ng/kg/min; Calbiochem, 2787322) were surgically implanted subcutaneously in the mid-scapular area over the shoulder blade followed by closing the wound with clips. AAA formation was analyzed after 14 or 28 days of Ang II infusion.

Topical Elastase/BAPN model was performed according to previously described protocol^35^. Ten microliters (7.6 mg protein/ml, 4 U/mg) of active elastase (Sigma Aldrich, 1002730705) or 10 μl of heat de-activated (100 °C for 30 min) elastase (sham) was applied on top of the infrarenal abdominal aorta of C57BL/6 or *Il27ra*^*−/−*^ mice for 5 min followed by washing of the exposed area with saline solution. Mice were given drinking water containing 0.2% BAPN fumarate salt (Sigma Aldrich, A3134) starting 2 days prior the surgery and elastase treatment until the end of study (day 14 after Elastase treatment), when AAA development was analyzed.

### Blood pressure measurements

Systolic blood pressure was measured on conscious mice using tail cuff system (CODA Non-Invasive Blood Pressure Monitor, Kent Scientific Corporation) 4 weeks after infusion of Ang II, according to the manufacturer’s protocol. Briefly, mice were placed in the holder maintained on the Animal Warming Platform. “Occlusion Cuff” and “VPR Cuff” were placed near the base of the tail. The cuffs were attached to the CODA Controller. Twenty cycles of measurement were performed and accepted cycles were automatically displayed in the spreadsheet format within the CODA application. Data were processed in Microsoft Excel by calculation of the average and standard deviation.

### Histology and immunofluorescence

For histological analysis, suprarenal aortas (with or without AAA) were isolated and embedded in Tissue-Tek O.C.T. (Optimal Cutting Temperature) compound (Sakura Finetek) and stored at −80 °C. Five micrometer serial frozen sections were cut and stained with hematoxylin (Sigma, SLBX9844) and eosin (ThermoScientific, 287569). All images were acquired with Nikon Eclipse 80i microscope.

Immunofluorescence staining was performed as previously described^68^. Five micrometer frozen sections of suprarenal aortas, containing AAA lesions, were fixed in cold acetone for 10 min followed by fixation in 1% paraformaldehyde in 100 mM dibasic sodium phosphate containing 60 mM lysine and 7 mM sodium periodate at pH 7.4 on ice. After that, sections were blocked with avidin/biotin blocking kit (Vector Laboratories) for 10 min, followed by blocking with 5% normal goat serum/1% bovine serum albumin in PBS for 15 min. Sections were stained at 4 °C overnight with primary rat anti-mouse CD11b-fluorescein isothiocyanate (FITC) (M1/70; BD Bioscience, 553310) and rat anti-mouse Ly6G-FITC (1A8; Biolegend, 127606) antibody in a 1:100 dilution, followed by staining with goat anti-FITC AlexaFluor 488 (Invitrogen, A-11096) secondary antibody in 1:1500 for 1 h at room temperature (RT). Sections were counterstained with 4′,6-diamidino-2-phenylindole (DAPI) (1 μg/ml) (Sigma, 32670) and mounted with Prolong Gold (Invitrogen, P36930). Images were examined on Leica SP8 DM6000 confocal microscope using HCX PLADO ×20 and ×40 oil-immersion objectives at 488 and 405 for DAPI excitation wavelengths and analyzed by Imaris (Bitplane).

### Verhoeff-Van Gieson staining of elastic fibers

Frozen, 5 μm sections of suprarenal aortas (with or without AAA) were cut and staining of elastic fibers was performed. Frozen sections were hydrated followed by staining in Verhoeff’s solution for 1 h. Afterwards, slides were incubated in 2% ferric chloride for 2 min and treated with 5% sodium thiosulfate for 1 min. Sections were counterstained in Van Gieson’s solution for 5 min and dehydrated (all reagents were from Electron Microscopy Sciences). Images were acquired with Nikon Eclipse 80i microscope.

### Peripheral blood cellularity

After killing, peripheral blood was collected by cardiac puncture to K_2_EDTA BD Microcontainer (BD Biosciences). White blood counts were obtained by VetScan HM5 analyzer (Abaxis).

### Flow cytometry

Cells were isolated from the aortas (with or without AAA or aortic arch) and spleen from mice infused with Ang II and were analyzed by flow cytometry. Mice were killed by CO_2_ inhalation and the aortas were perfused with PBS containing 2% heparin to remove all traces of blood. Suprarenal aortas were isolated, cut into small pieces, and incubated in a cocktail of digestion enzymes containing hyaluronidase (120 U/ml) (Sigma, H3506), collagenase I (450 U/ml) (Sigma, C0130), collagenase XI (250 U/ml) (Sigma, C7657), and DNAse I (120 U/ml) (Sigma, D4263) in Hank’s buffered salt solution (HBSS) for 55 min at 37 °C with gentle shaking. After incubation, cell suspensions were filtered through a 70 μm cell strainer and were stained with CD45-PerCP (30-F11; Biolegend, 103130), CD11b-PacBlue (M1/70; eBioscience, 101224), CD11c-APC (N418; Biolegend, 117310), Ly6G-Cy7APC (1A8; Biolegend, 127624), Ly6C-Cy7PE (HK1.4; Biolegend, 128018), TCRβ-PE (H57–597, Biolegend, 109208), and LIVE/DEAD Fixable Yellow Dead Cell dye (Life Technologies, 1862666), and analyzed on BD LSR II flow cytometer.

Blood was collected by cardiac puncture and erythrocytes were lysed by incubation with red blood cell (RBC) lysis buffer (15 mM NH_4_Cl, 0.1 mM NaHCO_3_, 0.1 mM Na_2_-EDTA) for 5 min at RT and washed with PBS. The spleen and blood were stained with the above mentioned antibody mix for mature immune cells.

For BM, bones (femurs and tibia) were isolated from mice implanted with Ang II or PBS-containing pumps for 4 weeks. BM was flushed with cold 2% fetal bovine serum (FBS) HBSS Ca^2+^- and Mg^2+^-free solution and filtered via a 70 μm cell strainer and erythrocytes were lysed by incubation with RBC lysis buffer (15 mM NH_4_Cl, 0.1 mM NaHCO_3_, 0.1 mM Na_2_-EDTA) for 5 min at RT. Cells were stained for HSPC subpopulation markers and analyzed by flow cytometry (BD LSR II). Live cells were defined as LIVE/DEAD negative. CD3-biotin (145–2C11; Biolegend, 100304), CD4-biotin (RM4-5; Biolegend, 100404), CD8a-biotin (53–6.7; Biolegend, 100704), CD19-biotin (6D5; Biolegend, 115504), B220-biotin (RA3-6B2; Biolegend, 103204), Gr1-biotin (RB6-8C5; Biolegend, 108404), CD11b-biotin (M1/70, Biolegend, 101204), CD11c-biotin (N418, eBioscience, 4272690), and Ter119-biotin (TER-119; Biolegend, 116204) antibody followed by Streptavidin Cy5PE (Biolegend, 405205) staining were used to define mature lineages. HSPCs were stained using c-kit-APC (2B8; BD Biosciences, 4299768), Sca-1-Cy7PE (D7; eBioscience, 4323278), CD150-PE (9D1; invitrogen, 1966373), CD48^−^PacBlue (HM48-1; Biolegend, 103418), CD34-FITC (RAM34; eBioscience, 4310179), and CD16/32-Cy7APC (93; eBioscience, 101328).

All antibody were used at 1:50 dilution and LIVE/DEAD Fixable Yellow Dead Cell dye at 1:200 dilution.

To sort specific HSPC populations, FACS Aria II cell sorter (BD Biosciences) was used.

Data were analyzed using Flowjo Software (Version 9.7.6).

### In vivo cell trafficking

Blood was collected from *Apoe*^*−/−*^ CD45.1 and *Apoe*^*−/−*^*Il27ra*^*−/−*^ CD45.2 mice, and erythrocytes were lysed by incubation with RBC lysis buffer (15 mM NH_4_Cl, 0.1 mM NaHCO_3_, 0.1 mM Na_2-_EDTA) for 5 min at RT and washed with PBS. *Apoe*^*−/−*^ CD45.1 cells were labeled with 2.5 μM eFluor 670 dye (Invitrogen, 65–0840) and *Apoe*^*−/−*^*Il27ra*^*−/−*^ CD45.2 cells were labeled with 2.5 μM CellTracker^TM^ orange CMRA dye (Invitrogen, C34551) for 10 min at 37 °C, followed by washing with 10% FBS/Dulbeccoʼs phosphate-buffered saline (PBS). The labeled cells were mixed in 1:1 ratio and 1 × 10^6^ of total blood leukocytes were injected into *Apoe*^*−/−*^ recipient mice infused with Ang II pump for 3 days. The recruitment of immune cells into suprarenal aorta was assessed 48 h after cell transfer by FACS on single-cell suspensions of digested aortas.

### In vivo BrdU incorporation and cell cycle analysis

Mice were intraperitoneally injected with a single dose of BrdU at 1 mg/mouse (BD Biosciences) in sterile PBS. Bones were collected 4 h after injection and BrdU incorporation was assessed by FACS (BD LSR II) using BrdU Flow Kit (BD Biosciences, 7222631) according to the manufacturer’s protocol. Data were analyzed using Flowjo Software (Version 9.7.6). BM cells were stained for HSPC subpopulations markers and LIVE/DEAD staining was applied to exclude dead cells. Next, fixation in BD Cytofix/Cytoperm Buffer for 20 min was performed; after that, samples were incubated in BD Cytoperm Plus Buffer for 10 min on ice followed by incubation in BD Cytofix/Cytoperm Buffer for 5 min on ice and treated with DNase to expose incorporated BrdU for 1 h at 37 °C. Cells were stained with anti-BrdU-PerCP (3D4; BD Biosciences, 560808) antibody at 1:10 dilution for 20 min at RT. For cell cycle analysis, cells were additionally stained with 5 μg/ml of DAPI for 15 min at RT and analyzed by flow cytometry.

### Competitive BM reconstitution

*Apoe*^*−/−*^ CD45.1 recipient mice were irradiated in two doses of 550 rad each (for a total of 1100 rad), 4 h apart. Femurs and tibias of donor mice (*Apoe*^*−/−*^ CD45.1 or *Apoe*^*−/−*^*Il27ra*^*−/−*^ CD45.2) were collected, flushed with sterile PBS, and filtered through a 70 μm cell strainer. Donor mixes were prepared by mixing *Apoe*^*−/−*^ CD45.1 and *Apoe*^*−/−*^*Il27ra*^*−/−*^ CD45.2 BM cells in a 90%:10%, 10%:90%, and 50%:50% ratio, and resuspended in sterile PBS. Two hundred and fifty microliters of total BM suspension containing 5 × 10^6^ cells were injected i.v. into irradiated recipient mice. After BMT, recipient mice were maintained on antibiotics containing water for 2 weeks. The reconstitution efficiency was analyzed by FACS 4 weeks after BMT using CD45.1-APC (A20; eBioscience, 4339672) and CD45.2-PerCP (104; Biolegend, 109826) antibody in a 1:30 dilution. One month after reconstitution, mice were placed on WD for 8 weeks followed by PBS or Ang II pump subcutaneous implantation. BM, spleen, blood, suprarenal aorta, and aortic arch were collected and analyzed by FACS analysis 4 weeks later.

### Immunomagnetic isolation of HSPCs

HSPCs were isolated using EasySep Mouse Hematopoietic Progenitor Cell Isolation Kit (STEMCELL Tech, 19856 A) according to the manufacturer’s protocol. Briefly, BM cells from *Apoe*^*−/−*^, *Apoe*^*−/−*^*Il27ra*^*+/−*^, and *Apoe*^*−/−*^*Il27ra*^*−/−*^ mice were incubated for 15 min at 4 °C with hematopoietic progenitor cells biotin isolation cocktail followed by incubation with Streptavidin RapidSpheres for 10 min at 4 °C. Tubes with cell suspension were placed into the magnet and incubated for 3 min followed by pouring the enriched cell suspension. Collected fraction represented the enriched lineage negative cell fraction (HSPCs). After isolation, HSPCs were counted and used for gene expression, western blotting, or colony-formation assays.

### Colony-formation assay

HSPCs from *Apoe*^*−/−*^*, Apoe*^*−/−*^*Il27ra*^*+/−*^, and *Apoe*^*−/−*^*Il27ra*^*−/−*^ mice infused with Ang II were sorted using HSPC isolation kit (STEMCELL Tech, 19856 A) and plated at 1 × 10^3^ cells in 1 ml of MethoCult GF M3434 medium (STEMCELL Tech, 03434) according to the manufacturer’s instructions. Ang II (1 μg/ml; Calbiochem, 2787322), 25 ng/ml of recIL-27 (Invitrogen, 1960008), 200 nM of Ruxolitinib (Cayman Chemical, 116091), or their combination was used for in vitro stimulation. Total numbers of colonies were scored at day 6 using the light microscope (Nikon Eclipse).

### Western blotting

HSPCs were pelleted by centrifugation for 5 min at 3000 × *g* at 4 °C and lysed in RIPA (100 μL for 1 × 10^6^ cells) followed by centrifugation at 15,000 × *g* for 10 min at 4 °C to pellet the cell debris. The supernatant was collected for the analysis. Protein concentration was determined by Bicinchoninic Acid Protein Assay Kit (Sigma, 1001491004) according to the manufacturer’s protocol. Ten to 20 μg of cell lysates were separated by 4–20% Tris-glycine gels (Mini-PROTEAN TGX gels, Bio-Rad) and transferred to polyvinylidene difluoride membranes (Trans-Blot Turbo Transfer Pack, Bio-Rad). Each membrane was washed with TBST (10 mM Tris-HCl (pH 7.6), 150 mM NaCl, 0.1% Tween-20) and blocked with 5% skimmed milk in TBST for 1 h followed by overnight incubation at 4 °C with appropriate primary antibody: p21 (F-5; Santa Cruz Biotechnology, sc-6246) and p53 (OP33; EMD Millipore, OP33–100UG) at 1:500 dilution. Loading was evaluated by staining with β-actin-horseradish peroxidase (HRP) (Abcam, ab49900) antibody (1:100000) for 1 h at RT. Each membrane was washed and primary antibodies (except β-actin-HRP) were detected with a 1:5000 dilution of HRP-conjugated rabbit anti-mouse IgG (Cell Signaling, 7076S) or mouse anti-rabbit IgG (Cell Signaling, 7074S). The reactive bands were developed using ECL Prime western blotting detection reagent (Amersham) and were visualized with an autoradiography film (Lab Scientific).

### RNA isolation and gene expression

Suprarenal aortas/AAA were isolated from *Apoe*^*−/−*^*Il27ra*^*+/*−^ and *Apoe*^*−/−*^*Il27ra*^*−/−*^ mice implanted with Ang II pumps and homogenized in TRIzol reagent (Invitrogen, 15596018) with 2.8 mm ceramic beads (OMNI International, 19–646–3) using Bead Ruptor homogenizer. Total RNA was extracted using Aurum Total RNA Fatty and Fibrous Tissue Kit (Bio-Rad, 7326870) according to the manufacturer’s instructions. HSPCs were lysed in RLT Plus buffer (Qiagen, 157030074) and total RNA was isolated using RNeasy Plus Mini Kit (Qiagen, 74136) according to the manufacturer’s protocols. Complementary DNA was synthesized using iScript Reverse Transcription Supermix (Bio-Rad, 1708841) with random primers according to the manufacturer’s protocol. Q-RT-PCR was performed with CFX 96 connect Real-Time PCR Detection System (Bio-Rad) using iTaq Universal SYBR Green Supermix (Bio-Rad, 1725124). The following primers were used: *Rpl32* (FW 5′*-*TTCCTGGTCCACAATGTCAA-3′ and REV 5′-GGCTTTTCGGTTCTTAGAGGA-3′)*, Ccl2* (FW 5′-ATTGGGATCATCTTGCTGGT-3′ and REV 5′-CCTGCTGTTCACAGTTGCC-3′), *Ccl5* (FW 5′- CCACTTCTTCTCTGGGTTGG-3′ and REV 5′-GTGCCCACGTCAAGGAGTAT-3′), *Mmp9* (FW 5′- CTGGACAGCCAGACACTAAAG-3′ and REV 5′- CTCGCGGCAAGTCTTCAGAG-3′), *Mmp2* (FW 5′-GCCCCGAGACCGCTATGTCCACT-3′ and REV 5′-GCCCCACTTCCGGTCATCATCGTA-3′), *Tnf* (FW 5′- AGGGTCTGGGCCATAGAACT-3′ and REV 5′- CCACCACGCTCTTCTGTCTAC-3′), *Il1b* (FW 5′- GGTCAAAGGTTTGGAAGCAG-3′ and REV 5′- TGTGAAATGCCACCTTTTGA-3′), *Mpo* (FW 5′- CTCCTCACCAACCGCTCC-3′ and REV 5′- TGCTCTCGAACAAAGAGGGT-3′), *Il3ra* (FW 5′- CTGGCATCCCACTCTTCAGAT-3′ and REV 5′- GGTCCCAGCTCAGTGTGTA-3′), *Csfr3r* (FW 5′- TGCACCCTGACTGGAGTTAC-3′ and REV 5′- TGAAATCTCGATGTGTCCACAG-3′), *Klf5* (FW 5′- CAGGCCACCTACTTTCCCC-3′ and REV 5′- GAATCGCCAGTTTGGAAGCAA-3′), *Myc (FW 5*′TCAAGAGGCGAACACACAAC-3′ and REV 5′- GGCCTTTTCATTGTTTTCCA-3′), and *Ccnd1* (FW 5′- GGGTGGGTTGGAAATGAAC-3′ and REV 5′- TCCTCTCCAAAATGCCAGAG-3′). Gene expression was normalized to *Rpl32* expression.

### RNA-sequencing and data processing and analysis

A total of 5000 LT-HSCs (Lin^−^Sca-1^+^c-kit^+^CD150^+^CD48^-^) or HPC-1 (Lin^−^Sca-1^+^c-kit^+^CD150^−^CD48^+^) were FACS-sorted from the BM of *Apoe*^*−/−*^*, Apoe*^*−/−*^*Il27ra*^*+/−*^, or *Apoe*^*−/−*^*Il27ra*^*−/−*^ mice infused with Ang II or PBS for 2 weeks. RNA was extracted using RNA Isolation Kit (Qiagen) according to the manufacturer’s protocol. Total RNA libraries were prepared by using Pico Input SMARTer Stranded Total RNA-Seq Kit (Takara). Total RNA (250 pg–10 ng) from each sample was reverse-transcribed via random priming. Full-length cDNA was obtained with SMART (Switching Mechanism At 5′-end of RNA Template) technology. The template-switching reaction keeps the strand orientation of the RNA. The ribosomal cDNA is hybridized to mammalian-specific R-Probes and then cleaved by ZapR. Libraries containing Illumina adapter with TruSeq HT indexes were subsequently pooled and loaded to the HiSeq 2500. Paired-end reads at 75 bp with 30 million reads per sample were generated for the bioinformatic analysis. RNA-sequencing data were aligned using STAR^69^ against mm10 genome and RSEM v1.2.12 software^70^ was used to estimate gene-level read counts using Ensemble transcriptome information. Only samples with at least 20% exonic reads considered by RSEM were used for analysis. DESeq2^71^ was used to estimate significance of differential expression difference between any two experimental groups with mouse gender used as an additional factor. Overall gene expression changes were considered significant if passed FDR < 5% thresholds unless stated otherwise. Putative regulator GSEA was done using Qiagen’s Ingenuity® Pathway Analysis software (IPA®, Qiagen Redwood City, www.qiagen.com/ingenuity) on genes that passed nominal *p* < 0.05 in comparison of Ang II in *Apoe*^*−/−*^*Il27ra*^*−/−*^ LT-HSCs vs. *Apoe*^*−/−*^*Il27ra*^*+/−*^ LT-HSCs using “Upstream Regulators” option. Upstream regulators with significantly predicted activation state (|*Z*-score| > 1.5) that in addition passed *p* < 0.05 target enrichment threshold with at least five target genes were reported. Reactome pathway enrichment analysis was done on gene list ranked by significance *p*-value and direction of change between Ang II in *Apoe*^*−/−*^*Il27ra*^*−/−*^ LT-HSCs vs Ang II in *Apoe*^*−/−*^ LT-HSCs using GSEA^51^ and pathways that passed *p* < 0.05 significance threshold were considered significant.

### Statistics

Student’s two-tailed *t*-test was used for comparison between two groups. Survival curve data were analyzed using the long-rank test. Fisher’s exact and Wilcoxon’s signed-rank tests were used to compare conditions when appropriate. Data were analyzed using the GraphPad Prism Software (Version 6.0). Data are presented as mean ± SEM; *p < 0.05, **p < 0.01, ***p < 0.001. A *p*-value < 0.05 was considered statistically significant.

### Reporting summary

Further information on research design is available in the Nature Research Reporting Summary linked to this article.

## Supplementary information

Supplementary Information

Reporting Summary

Source Data

## Data Availability

Sequence data are available at the Gene Expression Omnibus (GEO) accession number GSE129881. The source data underlying Figs. 1–5, 7 and Supplementary Figs. 1–3, 6–8, 10 are provided as Source Data file. The datasets generated and analyzed during the current study are available from the corresponding author upon reasonable request.

## References

[1] Nordon IM, Hinchliffe RJ, Loftus IM, Thompson MM (2011). Pathophysiology and epidemiology of abdominal aortic aneurysms. Nat. Rev. Cardiol..

[2] Dale MA, Ruhlman MK, Baxter BT (2015). Inflammatory cell phenotypes in AAAs: their role and potential as targets for therapy. Arterioscler. Thromb. Vasc. Biol..

[3] Shimizu K, Mitchell RN, Libby P (2006). Inflammation and cellular immune responses in abdominal aortic aneurysms. Arterioscler. Thromb. Vasc. Biol..

[4] Raffort J (2017). Monocytes and macrophages in abdominal aortic aneurysm. Nat. Rev. Cardiol..

[5] Peshkova IO, Schaefer G, Koltsova EK (2016). Atherosclerosis and aortic aneurysm - is inflammation a common denominator?. FEBS J..

[6] Daugherty A, Manning MW, Cassis LA (2000). Angiotensin II promotes atherosclerotic lesions and aneurysms in apolipoprotein E-deficient mice. J. Clin. Investig..

[7] Bruemmer D, Daugherty A, Lu H, Rateri DL (2011). Relevance of angiotensin II-induced aortic pathologies in mice to human aortic aneurysms. Ann. N. Y. Acad. Sci..

[8] Liu J (2015). Associations of ApoAI and ApoB-containing lipoproteins with AngII-induced abdominal aortic aneurysms in mice. Arterioscler. Thromb. Vasc. Biol..

[9] Mochizuki S (2007). Valsartan in a Japanese population with hypertension and other cardiovascular disease (Jikei Heart Study): a randomised, open-label, blinded endpoint morbidity-mortality study. Lancet.

[10] Sahar S (2005). Angiotensin II enhances interleukin^−^18 mediated inflammatory gene expression in vascular smooth muscle cells: a novel cross-talk in the pathogenesis of atherosclerosis. Circ. Res..

[11] Pueyo ME (2000). Angiotensin II stimulates endothelial vascular cell adhesion molecule-1 via nuclear factor-kappaB activation induced by intracellular oxidative stress. Arterioscler. Thromb. Vasc. Biol..

[12] Forrester SJ (2018). Angiotensin II signal transduction: an update on mechanisms of physiology and pathophysiology. Physiol. Rev..

[13] Rateri DL (2011). Endothelial cell-specific deficiency of Ang II type 1a receptors attenuates Ang II-induced ascending aortic aneurysms in LDL receptor-/- mice. Circ. Res..

[14] Poduri A (2015). Fibroblast angiotensin II type 1a receptors contribute to angiotensin II-induced medial hyperplasia in the ascending aorta. Arterioscler. Thromb. Vasc. Biol..

[15] Lu H, Rateri DL, Bruemmer D, Cassis LA, Daugherty A (2012). Novel mechanisms of abdominal aortic aneurysms. Curr. Atheroscler. Rep..

[16] Wang J (2014). IgE actions on CD4+ T cells, mast cells, and macrophages participate in the pathogenesis of experimental abdominal aortic aneurysms. EMBO Mol. Med..

[17] Manz MG, Boettcher S (2014). Emergency granulopoiesis. Nat. Rev. Immunol..

[18] Mellak S (2015). Angiotensin II mobilizes spleen monocytes to promote the development of abdominal aortic aneurysm in Apoe-/- mice. Arterioscler. Thromb. Vasc. Biol..

[19] Meher AK (2018). Novel role of IL (interleukin)-1beta in neutrophil extracellular trap formation and abdominal aortic aneurysms. Arterioscler. Thromb. Vasc. Biol..

[20] Eliason JL (2005). Neutrophil depletion inhibits experimental abdominal aortic aneurysm formation. Circulation.

[21] Borghesi L (2014). Hematopoiesis in steady-state versus stress: self-renewal, lineage fate choice, and the conversion of danger signals into cytokine signals in hematopoietic stem cells. J. Immunol..

[22] Glatman Zaretsky A, Engiles JB, Hunter CA (2014). Infection^−^induced changes in hematopoiesis. J. Immunol..

[23] Zhao JL, Baltimore D (2015). Regulation of stress-induced hematopoiesis. Curr. Opin. Hematol..

[24] Cortez-Retamozo V (2013). Angiotensin II drives the production of tumor-promoting macrophages. Immunity.

[25] Kim S (2016). Angiotensin II regulation of proliferation, differentiation, and engraftment of hematopoietic stem cells. Hypertension.

[26] Hunter CA, Kastelein R (2012). Interleukin-27: balancing protective and pathological immunity. Immunity.

[27] Yoshida H, Hunter CA (2015). The immunobiology of interleukin-27. Annu. Rev. Immunol..

[28] Meka RR, Venkatesha SH, Dudics S, Acharya B, Moudgil KD (2015). IL-27-induced modulation of autoimmunity and its therapeutic potential. Autoimmun. Rev..

[29] Hirase T (2013). Interleukin 27 inhibits atherosclerosis via immunoregulation of macrophages in mice. Am. J. Physiol. Heart Circ. Physiol..

[30] Koltsova EK (2012). Interleukin-27 receptor limits atherosclerosis in Ldlr-/- mice. Circ. Res..

[31] Peshkova IO, Fatkhullina AR, Mikulski Z, Ley K, Koltsova EK (2017). IL-27R signaling controls myeloid cells accumulation and antigen-presentation in atherosclerosis. Sci. Rep..

[32] Manning MW, Cassi LA, Huang J, Szilvassy SJ, Daugherty A (2002). Abdominal aortic aneurysms: fresh insights from a novel animal model of the disease. Vasc. Med..

[33] Lu H (2016). Hypercholesterolemia induced by a PCSK9 gain-of-function mutation augments angiotensin II-induced abdominal aortic aneurysms in C57BL/6 mice-brief report. Arterioscler. Thromb. Vasc. Biol..

[34] Daugherty A, Manning MW, Cassis LA (2001). Antagonism of AT2 receptors augments angiotensin II-induced abdominal aortic aneurysms and atherosclerosis. Br. J. Pharmacol..

[35] Lu G (2017). A novel chronic advanced stage abdominal aortic aneurysm murine model. J. Vasc. Surg..

[36] Boettcher S, Manz MG (2017). Regulation of inflammation- and infection-driven hematopoiesis. Trends Immunol..

[37] Oguro H, Ding L, Morrison SJ (2013). SLAM family markers resolve functionally distinct subpopulations of hematopoietic stem cells and multipotent progenitors. Cell Stem Cell.

[38] Cabezas-Wallscheid N (2014). Identification of regulatory networks in HSCs and their immediate progeny via integrated proteome, transcriptome, and DNA methylome analysis. Cell Stem Cell.

[39] Seita J, Weissman IL (2010). Hematopoietic stem cell: self-renewal versus differentiation. Wiley Interdiscip. Rev. Syst. Biol. Med..

[40] Yamada T, Park CS, Lacorazza HD (2013). Genetic control of quiescence in hematopoietic stem cells. Cell Cycle.

[41] Hrstka R (2016). AGR2 oncoprotein inhibits p38 MAPK and p53 activation through a DUSP10-mediated regulatory pathway. Mol. Oncol..

[42] Wilson A (2004). c-Myc controls the balance between hematopoietic stem cell self-renewal and differentiation. Genes Dev..

[43] Gui J, Zhao B, Lyu K, Tong W, Fuchs SY (2017). Downregulation of the IFNAR1 chain of type 1 interferon receptor contributes to the maintenance of the haematopoietic stem cells. Cancer Biol. Ther..

[44] Essers MA (2009). IFNalpha activates dormant haematopoietic stem cells in vivo. Nature.

[45] Chaves-Ferreira M (2016). The cyclin D1 carboxyl regulatory domain controls the division and differentiation of hematopoietic cells. Biol. Direct.

[46] Bhagat TD (2013). miR-21 mediates hematopoietic suppression in MDS by activating TGF-beta signaling. Blood.

[47] Sun W (2010). miR-223 and miR-142 attenuate hematopoietic cell proliferation, and miR-223 positively regulates miR-142 through LMO2 isoforms and CEBP-beta. Cell Res..

[48] Johnnidis JB (2008). Regulation of progenitor cell proliferation and granulocyte function by microRNA-223. Nature.

[49] Johnson CD (2007). The let-7 microRNA represses cell proliferation pathways in human cells. Cancer Res..

[50] Sampson VB (2007). MicroRNA let-7a down^−^regulates MYC and reverts MYC-induced growth in Burkitt lymphoma cells. Cancer Res..

[51] Subramanian A (2005). Gene set enrichment analysis: a knowledge-based approach for interpreting genome-wide expression profiles. Proc. Natl Acad. Sci. USA.

[52] Murphy AJ (2011). ApoE regulates hematopoietic stem cell proliferation, monocytosis, and monocyte accumulation in atherosclerotic lesions in mice. J. Clin. Invest..

[53] MacTaggart JN, Xiong W, Knispel R, Baxter BT (2007). Deletion of CCR2 but not CCR5 or CXCR3 inhibits aortic aneurysm formation. Surgery.

[54] Ohno T (2018). Cytokine profile of human abdominal aortic aneurysm: involvement of JAK/STAT pathway. Ann. Vasc. Dis..

[55] Xu J, Ehrman B, Graham LM, Eagleton MJ (2012). Interleukin-5 is a potential mediator of angiotensin II-induced aneurysm formation in apolipoprotein E knockout mice. J. Surg. Res..

[56] King VL (2009). Interferon-gamma and the interferon-inducible chemokine CXCL10 protect against aneurysm formation and rupture. Circulation.

[57] Sharma AK (2012). Experimental abdominal aortic aneurysm formation is mediated by IL-17 and attenuated by mesenchymal stem cell treatment. Circulation.

[58] Lareyre F (2017). TGFbeta (transforming growth factor-beta) blockade induces a human-like disease in a nondissecting mouse model of abdominal aortic aneurysm. Arterioscler. Thromb. Vasc. Biol..

[59] Johnston WF (2014). Inhibition of interleukin-1beta decreases aneurysm formation and progression in a novel model of thoracic aortic aneurysms. Circulation.

[60] Passegue E, Wagers AJ, Giuriato S, Anderson WC, Weissman IL (2005). Global analysis of proliferation and cell cycle gene expression in the regulation of hematopoietic stem and progenitor cell fates. J. Exp. Med..

[61] Sato T (2009). Interferon regulatory factor-2 protects quiescent hematopoietic stem cells from type I interferon^−^dependent exhaustion. Nat. Med..

[62] Nakamura-Ishizu A, Takizawa H, Suda T (2014). The analysis, roles and regulation of quiescence in hematopoietic stem cells. Development.

[63] van Kampen E, Jaminon A, van Berkel TJ, Van Eck M (2014). Diet-induced (epigenetic) changes in bone marrow augment atherosclerosis. J. Leukoc. Biol..

[64] Furusawa J (2016). Promotion of expansion and differentiation of hematopoietic stem cells by interleukin-27 into myeloid progenitors to control infection in emergency myelopoiesis. PLoS Pathog..

[65] Copley MR (2013). The Lin28b-let-7-Hmga2 axis determines the higher self-renewal potential of fetal haematopoietic stem cells. Nat. Cell Biol..

[66] Emmrich S (2014). miR-99a/100~125b tricistrons regulate hematopoietic stem and progenitor cell homeostasis by shifting the balance between TGFbeta and Wnt signaling. Genes Dev..

[67] Schultz J, Lorenz P, Gross G, Ibrahim S, Kunz M (2008). MicroRNA let-7b targets important cell cycle molecules in malignant melanoma cells and interferes with anchorage-independent growth. Cell Res..

[68] Koltsova EK (2012). Dynamic T cell-APC interactions sustain chronic inflammation in atherosclerosis. J. Clin. Investig..

[69] Dobin A (2013). STAR: ultrafast universal RNA-seq aligner. Bioinformatics.

[70] Li B, Dewey CN (2011). RSEM: accurate transcript quantification from RNA-Seq data with or without a reference genome. BMC Bioinformatics.

[71] Love MI, Huber W, Anders S (2014). Moderated estimation of fold change and dispersion for RNA-seq data with DESeq2. Genome Biol..

