# Supplementary material for: IL-27 receptor-regulated stress myelopoiesis drives abdominal aortic aneurysm development
Source: Nat Commun. 2019 Nov 6;10:5046. doi: 10.1038/s41467-019-13017-4 (PMC6834661; doi:10.1038/s41467-019-13017-4)
Supplement: Supplementary file 1 — Supplementary Information [file 41467_2019_13017_MOESM1_ESM.pdf]

Supplementary Information

IL-27 receptor-regulated stress myelopoiesis drives abdominal aortic aneurysm development

Peshkova et al.

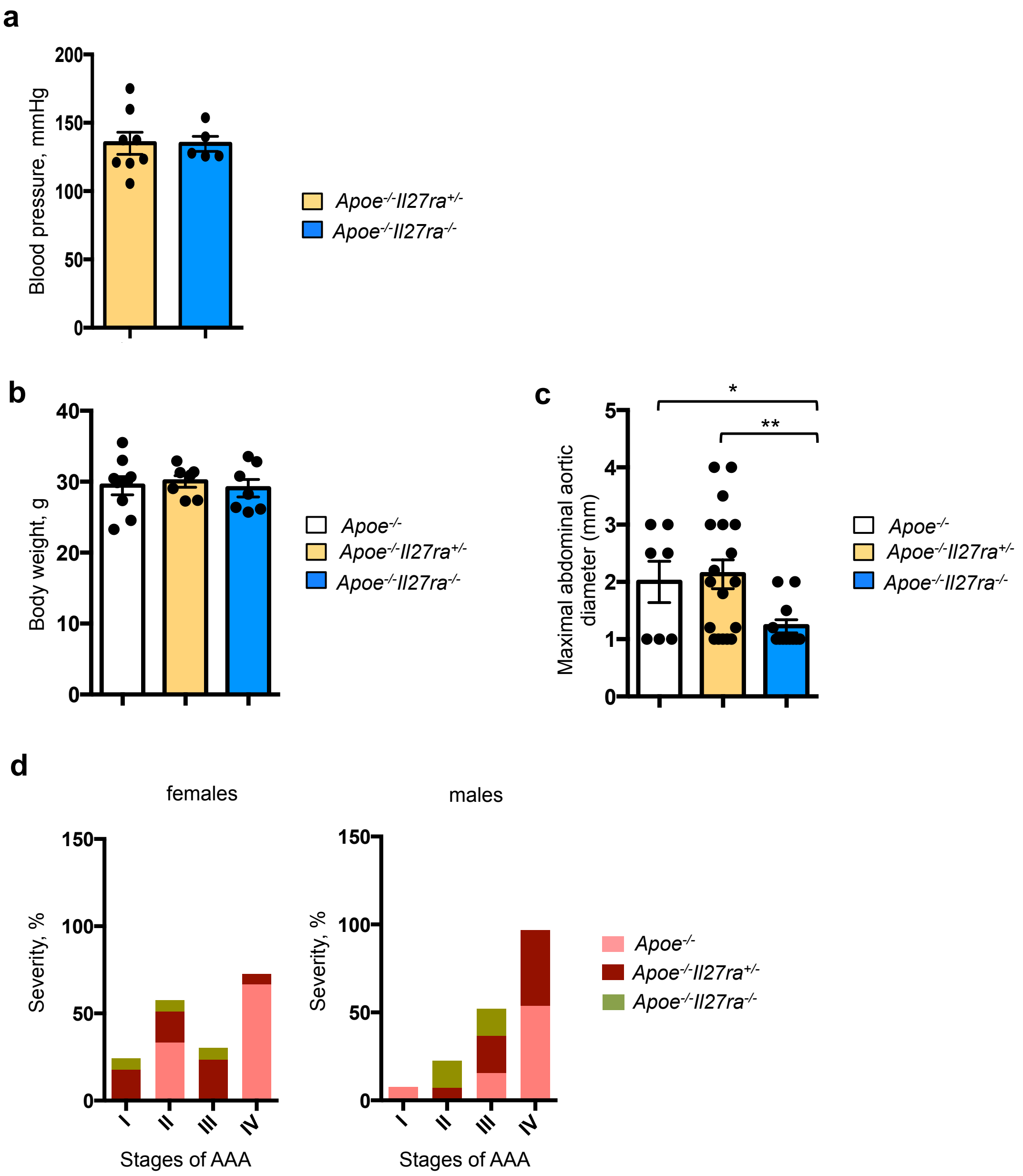

Supplementary Fig. 1. IL-27R deficiency does not affect blood pressure or body weight but affect AAA development. (a) Systolic blood pressure was measured using tail cuff system on conscious *Apoe*$^{-/-}$*Il27ra*$^{+/-}$ (n=8) and *Apoe*$^{-/-}$*Il27ra*$^{-/-}$ (n=5) mice infused with Ang II for 4 weeks. **(b)** Body weight of *Apoe*$^{-/-}$ (n=9), *Apoe*$^{-/-}$*Il27ra*$^{+/-}$ (n=7) or *Apoe*$^{-/-}$*Il27ra*$^{-/-}$ (n=7) mice after Ang II infusion. **(c)** Maximal abdominal aortic diameter of *Apoe*$^{-/-}$ (n=7) and *Apoe*$^{-/-}$*Il27ra*$^{+/-}$ (n=18) or *Apoe*$^{-/-}$*Il27ra*$^{-/-}$ (n=12) male and female mice infused with Ang II for 4 weeks. **(d)** Classification of AAA stages based on severity grade, immune infiltrate and elastin degradation in female and male mice. Data are mean ± SEM from 2 independent experiments. *p<0.05, **p<0.01, unpaired Student's *t* test (two-tailed).

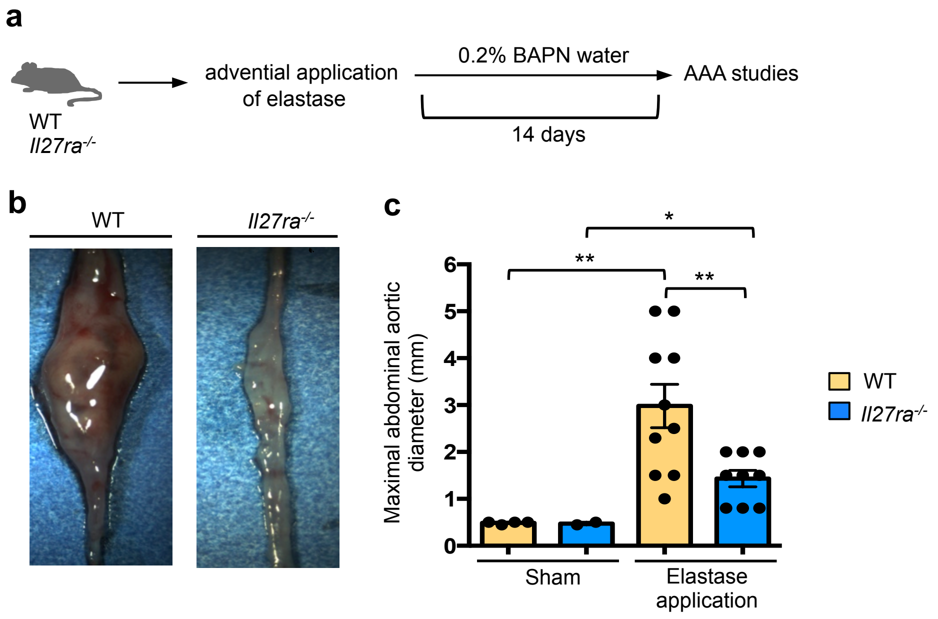

Supplementary Fig. 2. IL-27R deficiency protects from AAA development in Ang-II-independent (elastase +BAPN) model. (a) Scheme of the experiment. **(b)** Representative images of suprarenal aortas with developed AAA. **(c)** Maximal abdominal aortic diameter of sham operated WT (n=4) and *Il27ra*$^{-/-}$ (n=2) and elastase treated WT (n=10) and *Il27ra*$^{-/-}$ (n=9) male and female mice. Data are mean ± SEM from 2 independent experiments. *p<0.05, **p<0.01, unpaired Student's *t* test (two-tailed).

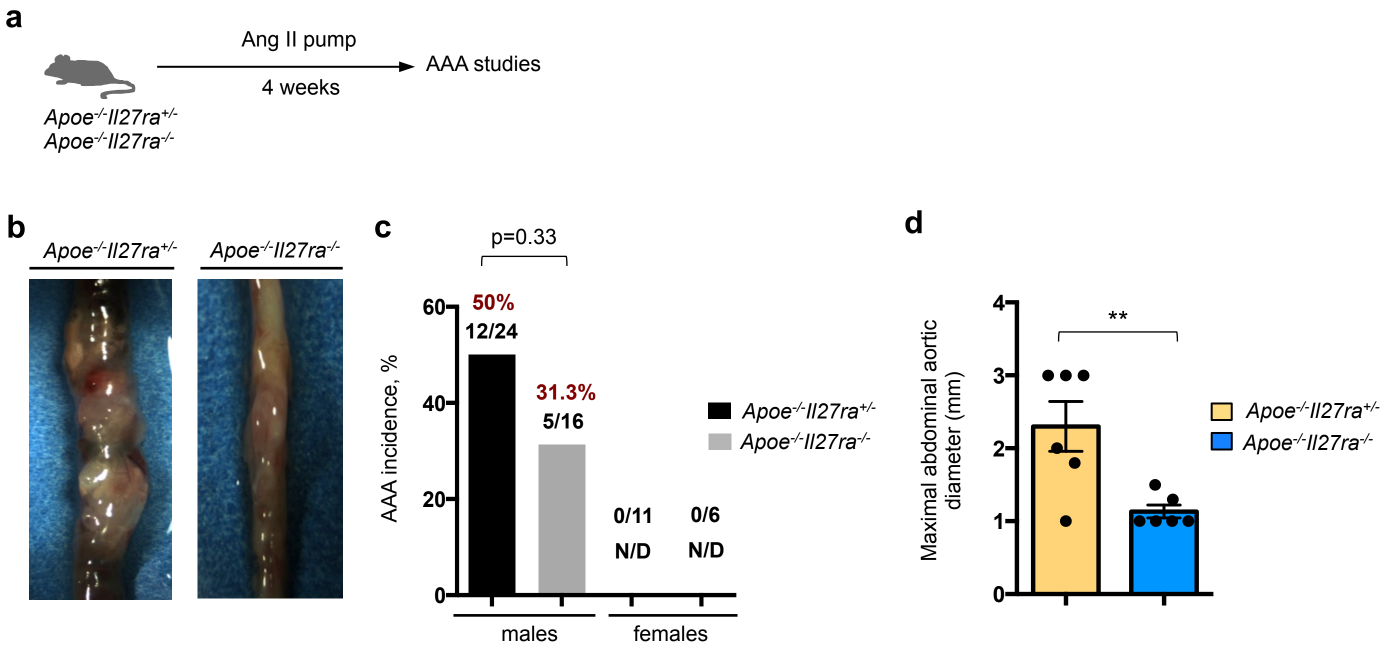

Supplementary Fig. 3. Ang II-induced AAA development in mice fed the chow diet. (a) Scheme of the experiment. *Apoe*$^{-/-}$*Il27ra*$^{+/-}$ and *Apoe*$^{-/-}$*Il27ra*$^{-/-}$ female and male mice were fed the CD for overall period of 12 weeks, where last 4 weeks of feeding they were implanted with pumps containing Ang II. **(b)** Representative images of suprarenal aortas with developed AAA. **(c)** Percentage of AAA incidence among *Apoe*$^{-/-}$*Il27ra*$^{+/-}$ (n=24) and *Apoe*$^{-/-}$*Il27ra*$^{-/-}$ (n=16) male and *Apoe*$^{-/-}$*Il27ra*$^{+/-}$ (n=11) and *Apoe*$^{-/-}$*Il27ra*$^{-/-}$ (n=6) female mice. Fisher's Exact Test (two sided) **(d)** Maximal abdominal aortic diameter of *Apoe*$^{-/-}$*Il27ra*$^{+/-}$ (n=6) and *Apoe*$^{-/-}$*Il27ra*$^{-/-}$ (n=6) male mice. Data are mean ± SEM from 2 independent experiments. **$p<0.01$, unpaired Student's *t* test (two-tailed).

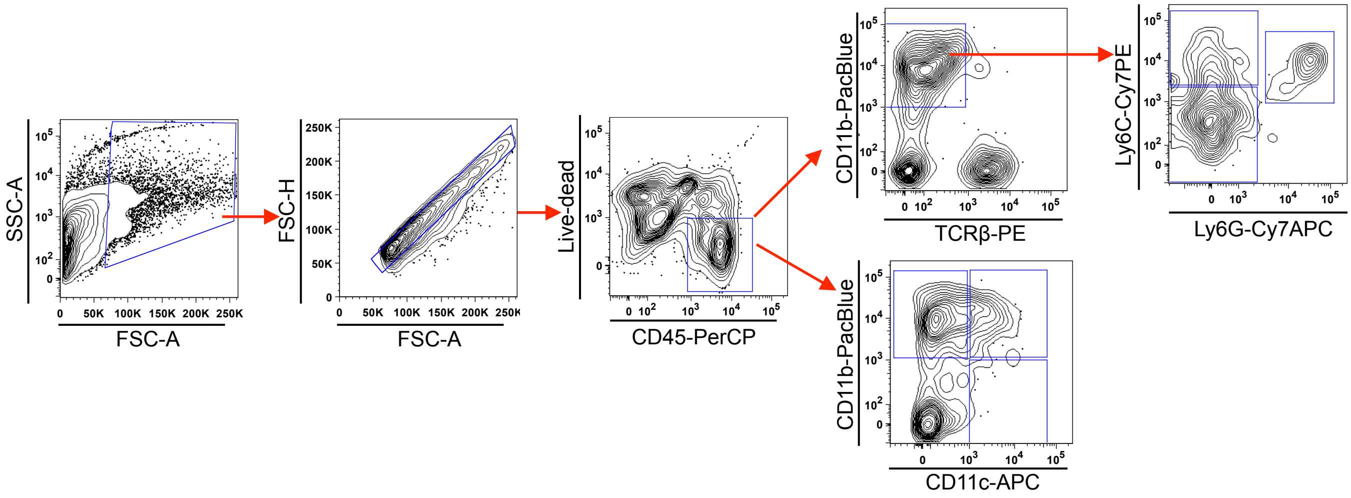

Supplementary Fig. 4. Flow cytometry gating strategy for FACS analysis employed to identify populations of mature immune cells.

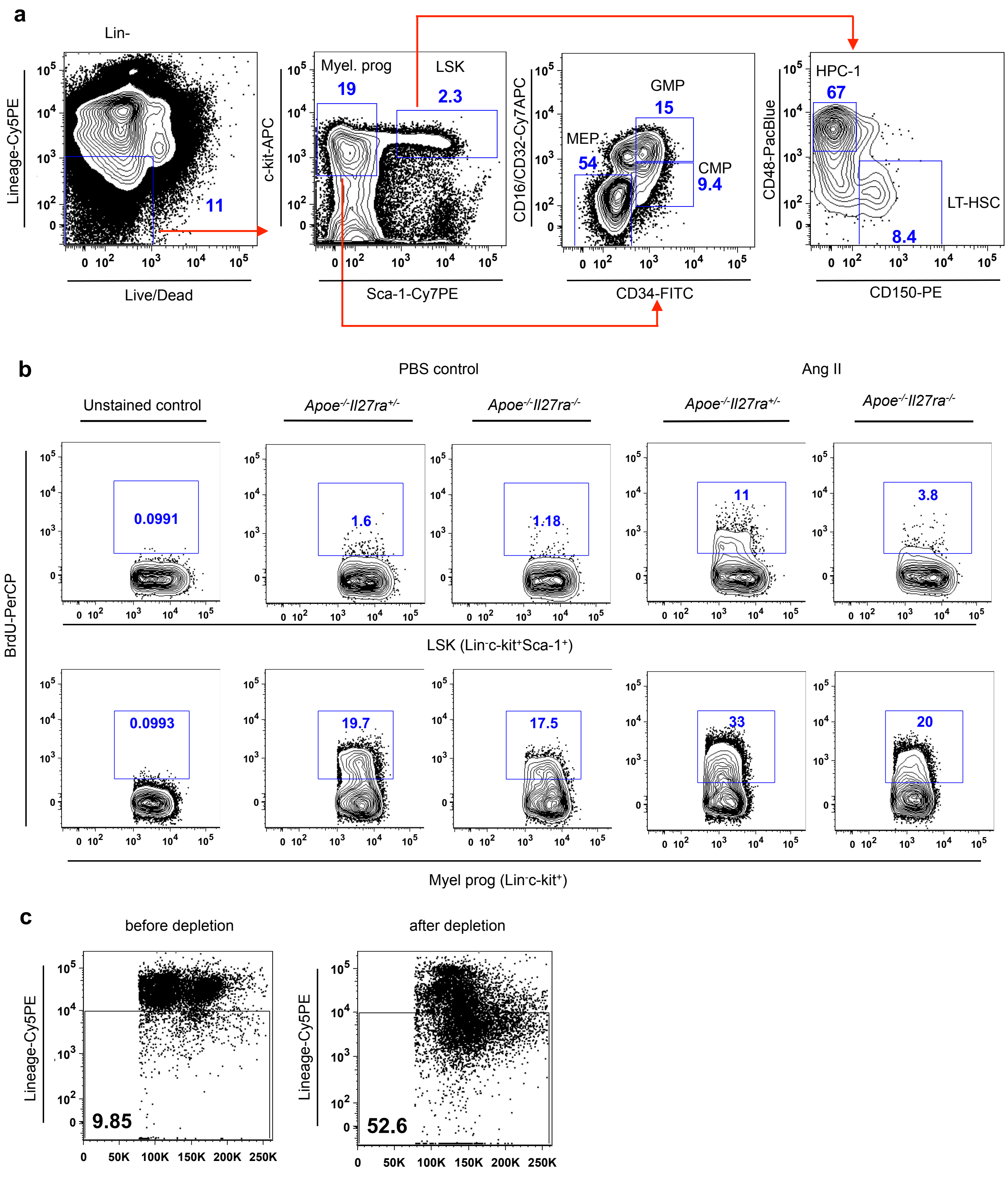

Supplementary Fig. 5. Flow cytometry gating strategy employed to identify populations of hematopoietic stem and progenitor cells. (a) Gating strategy used to identify LSK, including LT-HSC and HPC-1 and myeloid progenitors, including CMP, GMP and MEP populations. **(b)** BrdU^{+} cell populations (LSK and myeloid progenitors) as determined based on BrdU negative control. **(c)** Purity of HSPCs isolation by magnetic separation.

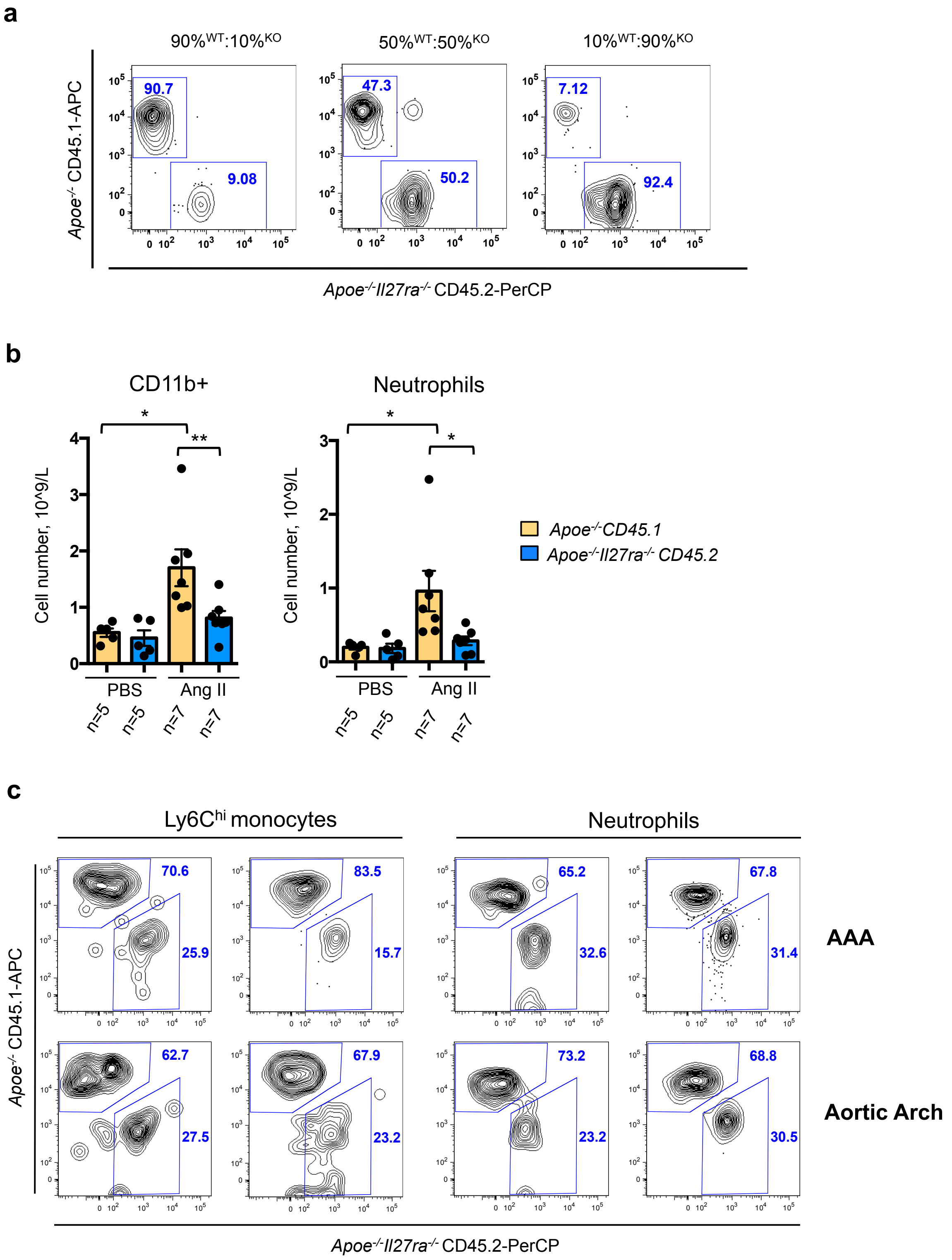

Supplementary Fig. 6. Competitive bone marrow transfer. (a) Bone marrow reconstitution efficiency was determined by flow cytometry in the peripheral blood of recipient mice 4 weeks after competitive bone marrow transplantation. Cell number of CD11b$^+$ cells and neutrophils (Ly6G$^+$) in blood after RBC lysis **(b)** and representative FACS plots of Ly6C^{hi+} monocytes and neutrophils (Ly6G$^+$) of CD45.1 (*Apoe*$^{-/-}$) and CD45.2 (*Apoe*$^{-/-}$*Il27ra*$^{-/-}$) origins in AAA and aortic arch **(c)** of recipient mice reconstituted with 50%*Apoe*$^{-/-}$:50%*Apoe*$^{-/-}$*Il27ra*$^{-/-}$ bone marrow mix followed by infusion with PBS or Ang II as determined by flow cytometry. Data are mean ± SEM from 2 independent experiments. *p<0.05, **p<0.01, unpaired Student's *t* test (two-tailed).

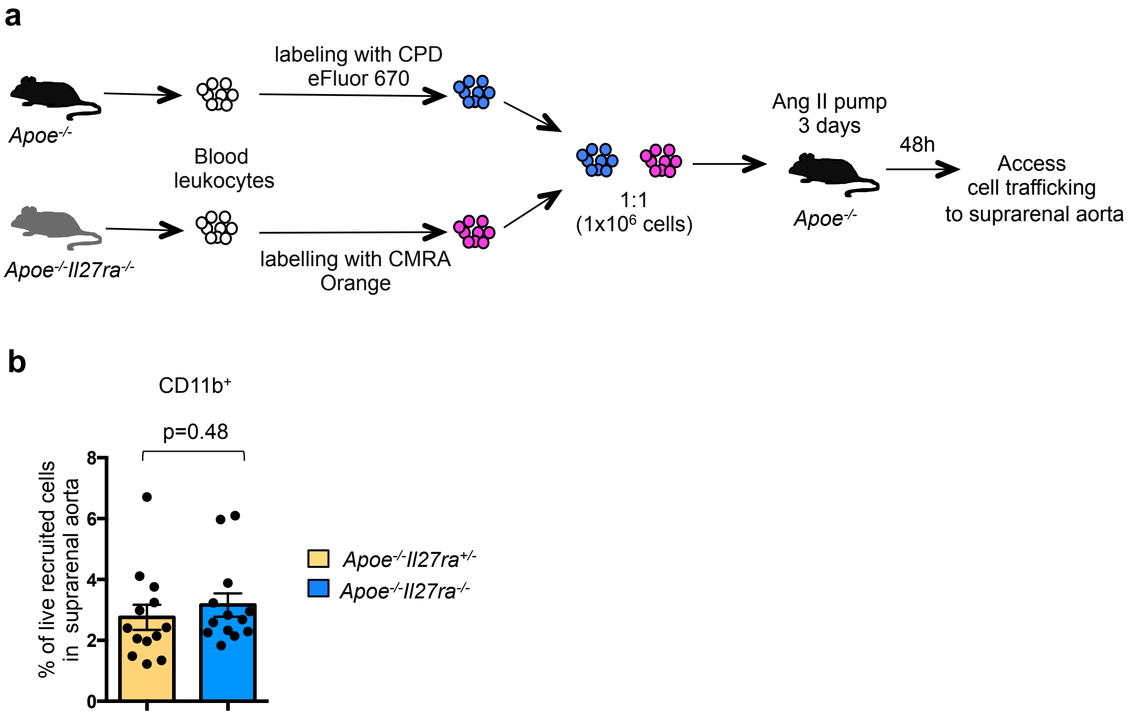

Supplementary Fig. 7. IL-27R deficiency does not affect trafficking of myeloid cells into suprarenal aorta during early stages of AAA development. (a) Scheme of the experiment. **(b)** Percentage of live recruited donor-specific $CD11b^+$ cells in suprarenal aorta of recipient mice (n=13). Data are mean ± SEM from 2 independent experiments. Unpaired Student's *t* test (two-tailed).

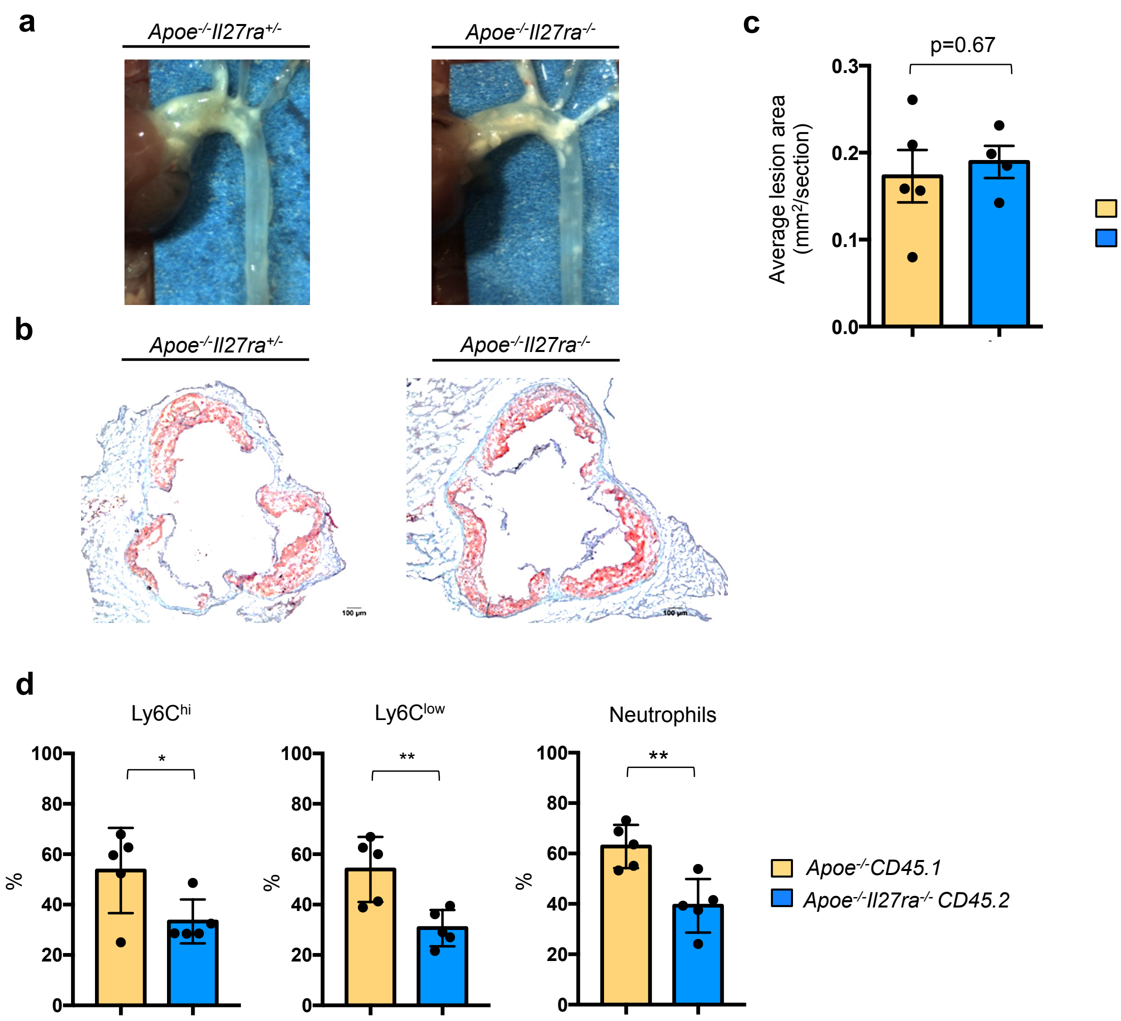

Supplementary Fig. 8. Ang II infusion accelerates atherosclerosis development in IL-27R sufficient, but not deficient mice. (a) Images of atherosclerotic lesions in aortic arch and **(b)** aortic root sections of *Apoe*$^{-/-}$*Il27ra*$^{+/-}$ and *Apoe*$^{-/-}$*Il27ra*$^{-/-}$ mice fed with WD for 12 weeks and infused with Ang II for last 4 weeks of feeding. **(c)** Quantitative comparison of aortic lesion size in *Apoe*$^{-/-}$*Il27ra*$^{+/-}$ (n=5) and *Apoe*$^{-/-}$*Il27ra*$^{-/-}$ (n=4) mice. Data are mean ± SEM from 2 independent experiments. (**d**) Proportion of live donor-specific Ly6C^{hi+}, Ly6C^{low+} monocytes and neutrophils (Ly6G^{+}) in aortic arch of recipient mice (n=5) reconstituted with donor mixes of 50%*Apoe*$^{-/-}$ CD45.1:50% *Apoe*$^{-/-}$*Il27ra*$^{-/-}$ CD45.2 total bone marrow cells and fed WD for 10 weeks, where last 2 weeks they were infused with Ang II. Data are mean ± SEM from 2 independent experiments. *$p<0.05$, **$p<0.01$, unpaired Student's *t* test (two-tailed).

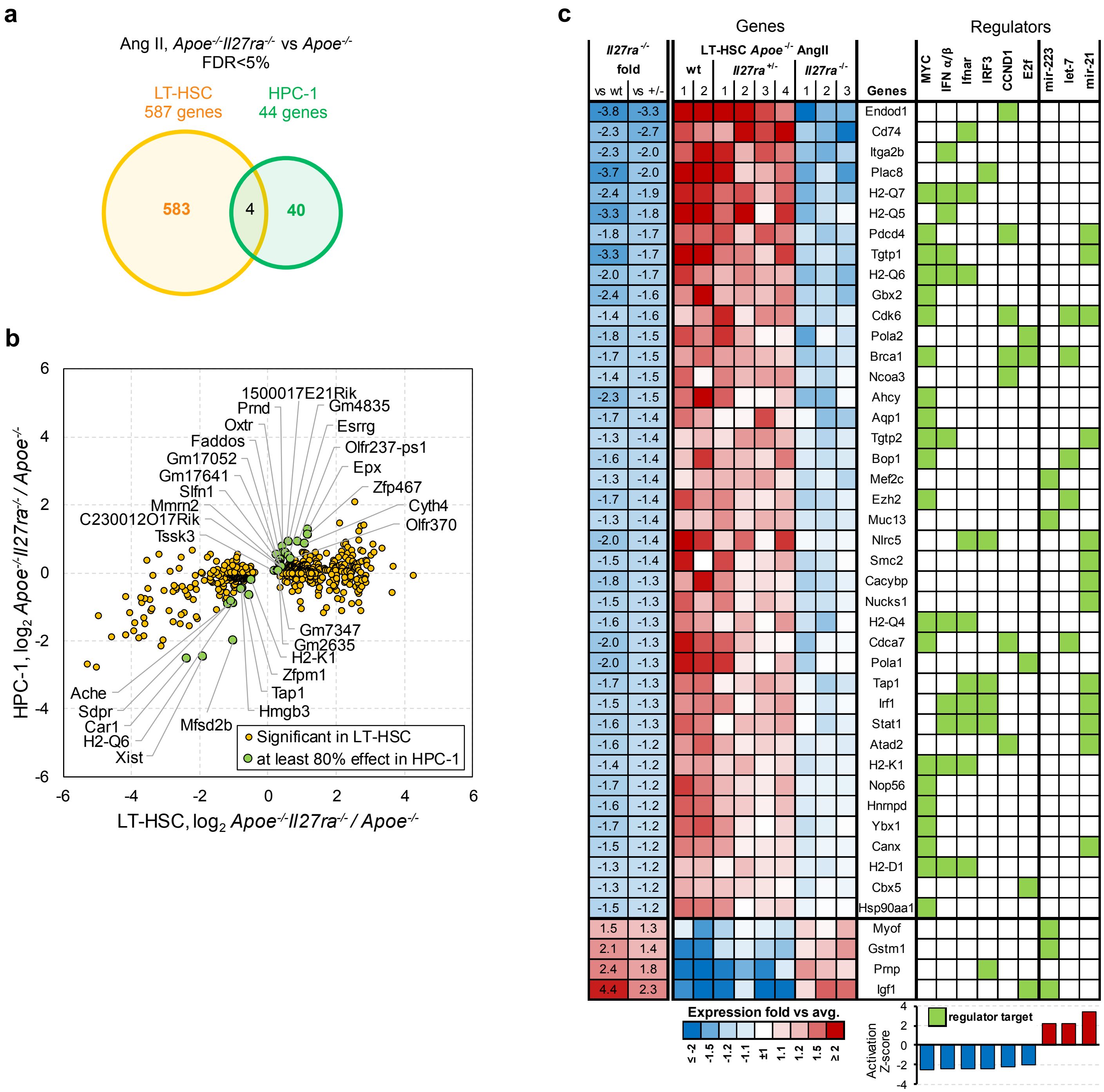

Supplementary Fig. 9. LT-HSCs (Lin$^-$Sca-1$^+$c-kit$^+$CD150$^+$CD48$^-$) and HPC-1 (Lin$^-$Sca-1$^+$c-kit$^+$CD150$^-$CD48$^+$) were FACS-sorted from bone marrow of *Apoe*$^{-/-}$ (n=2) , *Apoe*$^{-/-}$*Il27ra*$^{+/-}$ (n=4) or *Apoe*$^{-/-}$*Il27ra*$^{-/-}$ (n=3) mice fed WD for 12 weeks and infused with Ang II or PBS for last 2 weeks of feeding, followed by whole transcriptome analysis. **(a)** Overlap of genes between LT-HSC and HPC-1 obtained from IL-27R-sufficient and IL-27R-deficient *Apoe*$^{-/-}$ mice infused with Ang II, FDR<5%. **(b)** Only 29 genes of all 587 genes affected in LT-HSC had at least 80% similar effect in HPC-1. **(c)** Expression heatmap for significantly changed genes (p<0.05) between *Apoe*$^{-/-}$*Il27ra*$^{-/-}$ vs *Apoe*$^{-/-}$*Il27ra*$^{+/-}$ and *Apoe*$^{-/-}$ mice, known to be regulated by the 9 significantly enriched (p<0.05) regulators. Green squares indicate known regulator->target relationship from published literature as recorded in Ingenuity Knowledgebase. p<0.05, unpaired Student's *t* test (two-tailed).

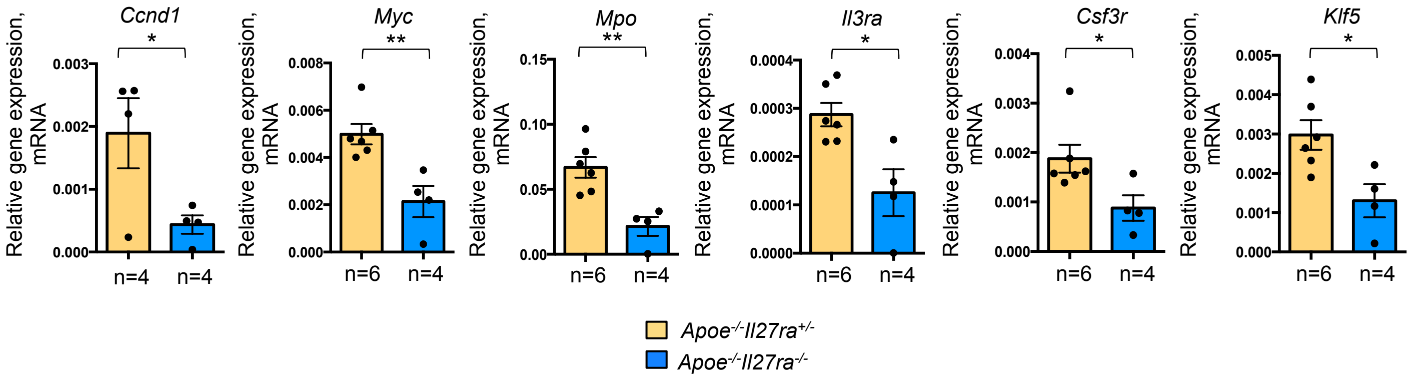

Supplementary Fig. 10. Decreased expression of genes regulating proliferation and myeloid lineage commitment in HSPCs of *Apoe$^{-/-}$Il27ra$^{-/-}$* mice. Relative gene expression in HSPCs isolated from WD-fed *Apoe$^{-/-}$Il27ra$^{+/-}$* or *Apoe$^{-/-}$Il27ra$^{-/-}$* mice infused with Ang II for 4 weeks were normalized to *Rpl32* gene expression and then normalized to gene expression in control *Apoe$^{-/-}$Il27ra$^{+/-}$* mice**.** Data are mean ± SEM from at least 2 independent experiments. *p<0.05, **p<0.01, unpaired Student's *t* test (two-tailed).
